# Neocortical synaptic engrams for remote contextual memories

**DOI:** 10.1038/s41593-022-01223-1

**Published:** 2022-12-23

**Authors:** Ji-Hye Lee, Woong Bin Kim, Eui Ho Park, Jun-Hyeong Cho

**Affiliations:** Department of Molecular, Cell and Systems Biology, University of California, Riverside, CA USA

**Keywords:** Long-term memory, Neural circuits, Long-term potentiation, Fear conditioning, Consolidation

## Abstract

While initial encoding of contextual memories involves the strengthening of hippocampal circuits, these memories progressively mature to stabilized forms in neocortex and become less hippocampus dependent. Although it has been proposed that long-term storage of contextual memories may involve enduring synaptic changes in neocortical circuits, synaptic substrates of remote contextual memories have been elusive. Here we demonstrate that the consolidation of remote contextual fear memories in mice correlated with progressive strengthening of excitatory connections between prefrontal cortical (PFC) engram neurons active during learning and reactivated during remote memory recall, whereas the extinction of remote memories weakened those synapses. This synapse-specific plasticity was CREB-dependent and required sustained hippocampal signals, which the retrosplenial cortex could convey to PFC. Moreover, PFC engram neurons were strongly connected to other PFC neurons recruited during remote memory recall. Our study suggests that progressive and synapse-specific strengthening of PFC circuits can contribute to long-term storage of contextual memories.

## Abstract (web summary)

Lee et al. show that the long-term storage of remote contextual memories involves progressive and synapse-specific strengthening of excitatory connections between memory engram neurons in the prefrontal cortex.

## Main

The acquisition of contextual memories requires hippocampal circuits. For instance, encoding of contextual fear memories involves synapse-specific plasticity in hippocampal CA3–CA1 and hippocampal–amygdala circuits^1,2^. Once acquired, contextual memories gradually mature to stabilized forms in the neocortex during systems-level memory consolidation^3–5^. The standard consolidation model proposes that the long-term storage of contextual memories may involve enduring synaptic changes in neocortical circuits^6,7^ such that remote memory recall depends less on the hippocampus^8^. However, synapse-specific substrates of remote contextual memories have not been identified.

Previous studies suggest that neurons in the medial prefrontal cortex (mPFC) and anterior cingulate cortex (ACC) have a pivotal role in the consolidation of remote but not recent contextual memories^9–11^. These prefrontal cortex (PFC) memory engram neurons are rapidly generated during learning, gradually mature with time and are reactivated during remote memory recall^12^. Although these studies identified neuronal correlates of remote contextual memories, how PFC engram neurons contribute to remote memory consolidation at the synaptic level remains poorly understood. Connections between PFC engram neurons may be strengthened during memory consolidation, synchronizing the activity of PFC engram neurons and facilitating their reactivation during remote memory recall. However, it remains to be determined whether and how the synaptic strength of neocortical circuits changes during systems consolidation. It is also unknown how PFC engram neurons are connected to other PFC neurons recruited during remote memory recall and those projecting to subcortical engram neurons or how systems consolidation affects these synapses. The transformation theory of systems consolidation proposes that an initially formed memory with contextual details remains dependent on the hippocampus and supports the development of a schematic memory with few contextual details in the neocortex^13,14^. However, how signals of hippocampal engram are conveyed to PFC for the maturation of neocortical engram remains incompletely understood. In this study, we demonstrated that remote memory consolidation involves progressive and synapse-specific strengthening of excitatory connections between PFC engram neurons, which requires sustained signals of hippocampal engram.

## Results

### Reactivation of mPFC engram neurons and remote memory recall

**Fig. 1:**
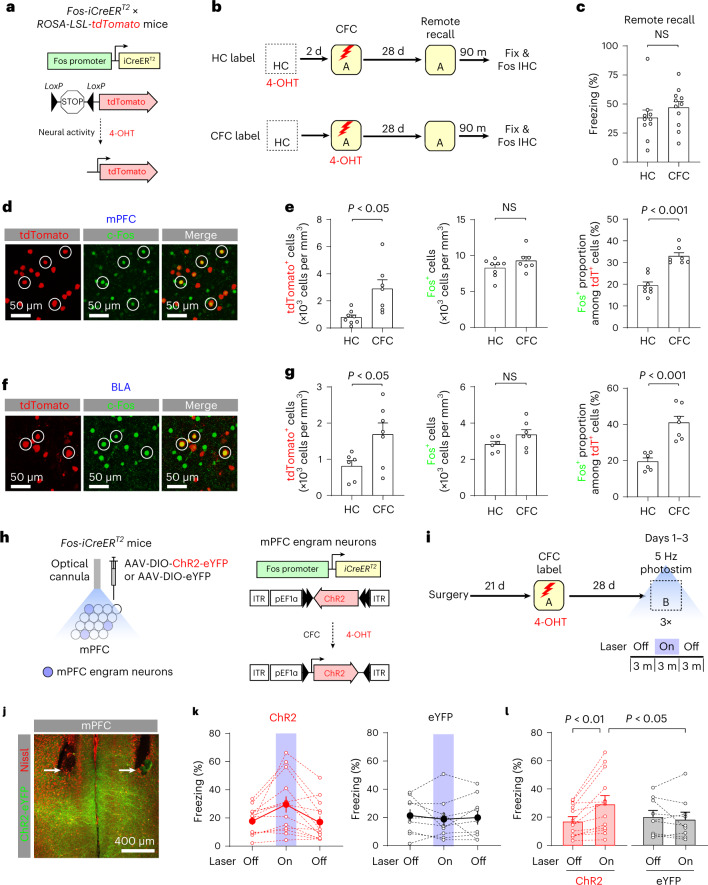
Activity-dependent labeling identified mPFC engram neurons, whose reactivation resulted in memory recall. **a,b**, Experimental setup for **c**–**g**. Active neurons expressed tdT in *Fos-iCreER*^*T2*^ × *ROSA-LSL-tdTomato* mice. Neurons active in the HC were labeled in the HC group (10 mice), whereas neurons active during CFC were labeled in the CFC group (11 mice). After remote memory recall test, brain tissues were immunolabeled for c-Fos. **c**, Freezing behavior during remote memory recall. NS, not significant. **d**, Images showing mPFC/PL neurons labeled with tdT (red) or c-Fos (green). Neurons labeled with both tdT and c-Fos are circled. **e**, Comparisons of the tdT^+^ cell density, c-Fos^+^ cell density and c-Fos^+^ proportion among all tdT^+^ neurons in the mPFC/PL. Unpaired *t* test (HC group: eight mice, CFC group: seven mice). **f**, Images showing BLA neurons labeled with tdT (red) or c-Fos (green). **g**, Comparisons of the tdT^+^ cell density, c-Fos^+^ cell density and c-Fos^+^ proportion among all tdT^+^ neurons in the BLA. Unpaired *t*-test (HC group: six mice, CFC group: seven mice). **h**, Experimental setup for (**i**–**l**). mPFC neurons active during CFC expressed ChR2-eYFP or eYFP. **i**, Four weeks after CFC, the mice received 5 Hz photostimulation in Context B. **j**, Image showing optical cannula tips (arrows) and ChR2-eYFP^+^ mPFC neurons (green). **k,l**, Summary plot showing the average freezing time in the presence and absence of photostimulation in the ChR2 (13 mice) and eYFP groups (nine mice). Repeated measures two-way ANOVA with post hoc comparisons (group × behavioral session interaction, *P* < 0.01). Data are presented as the mean ± s.e.m. Details of the statistical analyses are presented in Supplementary Tables 1 and 2. Source data

**Extended Data Fig. 1:**
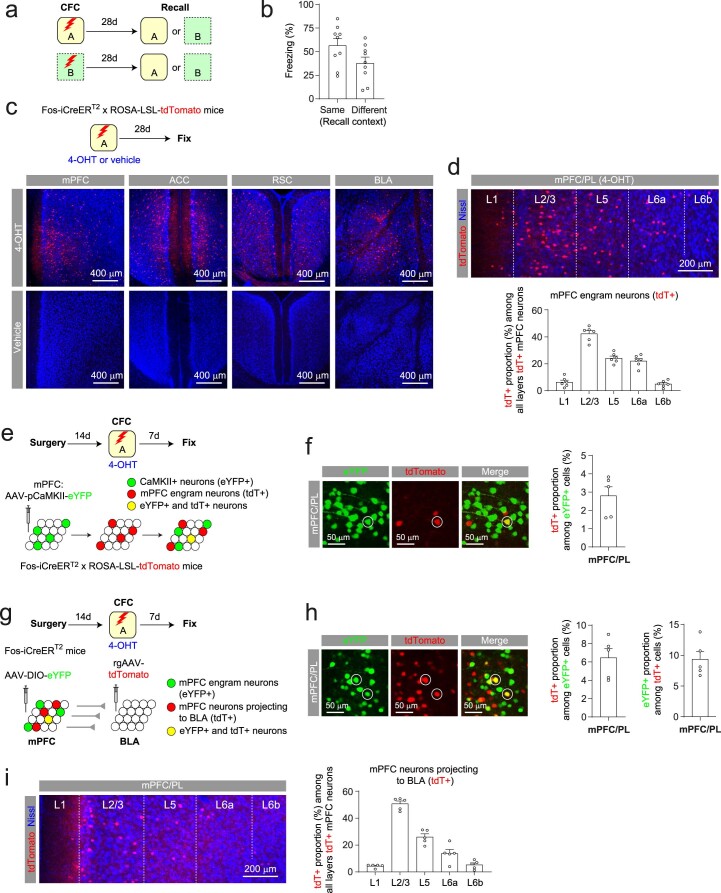
Labeling of mPFC engram neurons and their proportions among all CaMKII+ neurons and among all BLA projectors. (**a**) Mice were fear conditioned in Context A or B and tested for remote memory recall in the same (9 mice) or different context (9 mice). (**b**) Quantification of freezing behavior during remote memory recall as in (a). Mice tested in the same contexts tended to display more freezing behavior than mice tested in different contexts (*p* = 0.07, unpaired *t*-test). (**c**) Neurons active during CFC (engram neurons) were labeled with tdTomato (tdT). Images show tdT+ neurons (red) in mPFC, caudal anterior cingulate cortex (ACC), retrosplenial cortex (RSC), and basolateral amygdala (BLA) in mice that received 4-OHT but not vehicle injection after CFC (Blue: Nissl stain). (**d**) *Top*: tdT+ neurons (red) in different mPFC/PL layers in 4-OHT-injected mice in (c). *Bottom*: proportion of tdT+ neurons in each mPFC/PL layer among all tdT+ neurons (6 mice). (**e**) Experimental setup for (f). CaMKII+ mPFC pyramidal neurons expressed eYFP, whereas neurons active during CFC expressed tdT. (**f**) *Left*: eYFP+ (green) or tdT+ (red) mPFC/PL neurons. A both eYFP+ and tdT+ neuron is circled. *Right*: proportion of tdT+ neurons among all eYFP+ mPFC neurons (5 mice). (**g**) Experimental setup for (h)-(i). mPFC neurons projecting to BLA (BLA projectors) were retrogradely labeled with tdT. mPFC neurons active during CFC expressed eYFP. (**h**) *Left*: tdT+ (red) or eYFP+ (green) mPFC/PL neurons. Both tdT+ and eYFP+ neurons are circled. *Middle*: proportion of tdT+ neurons among all eYFP+ mPFC neurons. *Right*: proportion of eYFP+ neurons among all tdT+ mPFC neurons (5 mice). (**i**) *Left*: BLA projectors (tdT+, red) in different mPFC/PL layers. *Right*: proportion of BLA projectors in each mPFC/PL layer among all BLA projectors in mPFC (5 mice). Data are presented as the mean ± SEM. Source data

We used heterozygous *Fos-iCreER*^*T2*^ knock-in mice^15^ to label neurons recruited during contextual fear conditioning (CFC), in which the mice learn to associate a neutral context with aversive unconditioned stimuli (US; electric footshock) and display fear to the context. *Fos-iCreER*^*T2*^ × *ROSA-LSL-tdTomato* mice in the CFC group received the US in Context A and received 4-hydroxytamoxifen (4-OHT) injection (Fig. 1a,b). Neurons active during CFC expressed iCreER^T2^ under the control of c-Fos promoter, which induced the recombination of LoxP-STOP-LoxP sequence in the presence of 4-OHT, resulting in tdTomato (tdT) expression (Fig. 1a and Extended Data Fig. 1c). In the home cage (HC) group, neurons active in the HC were labeled with tdT and the mice were fear conditioned 2 d later. Four weeks after CFC, the mice displayed robust freezing behavior in Context A (Fig. 1c and Extended Data Fig. 1a,b). Neurons active during remote memory recall were immunolabeled for c-Fos. In the prelimbic (PL) division of the mPFC and basolateral amygdala (BLA), both tdT^+^ cell density and c-Fos^+^ proportion among all tdT^+^ neurons were higher in the CFC group than in the HC group, whereas c-Fos^+^ cell density did not differ between groups (Fig. 1d–g and Supplementary Table 1). These results suggest that CFC recruited mPFC/PL and BLA neurons, which were more likely reactivated during remote memory recall than neurons active in the HC. In the CFC group, tdT expression was highest in mPFC layer 2/3 and detected in 2.8 ± 0.5% of CaMKII^+^ neurons, while 6.5 ± 1.0% of tdT^+^ mPFC neurons projected to the BLA (mean ± s.e.m., five mice, Extended Data Fig. 1d–i).

We examined whether the reactivation of mPFC neurons active during CFC induced memory recall. After surgery for virus injection and optical cannula implantation, mice were fear conditioned in Context A to induce ChR2-eYFP or eYFP expression in mPFC neurons active during CFC (Fig. 1h–j). Four weeks after CFC, the mice were placed in Context B and received 5 Hz photostimulation, which substantially increased freezing behavior in the ChR2 group (laser off, 17.4 ± 2.9%; laser on, 29.6 ± 5.7%; mean ± s.e.m, 13 mice) but not in the eYFP group (laser off, 20.6 ± 4.2%; laser on, 18.7 ± 4.9%; 9 mice; Fig. 1k, l and Supplementary Table 2). Thus, the reactivation of mPFC neurons active during CFC induced fear in an irrelevant context. Our results suggest that a subset of mPFC neurons active during CFC was reactivated during remote memory recall, and optogenetic reactivation of these neurons induced memory recall. Thus, we termed these labeled mPFC neurons ‘mPFC engram neurons’^16^.

### Strengthening of PFC engram circuit in systems consolidation

**Fig. 2:**
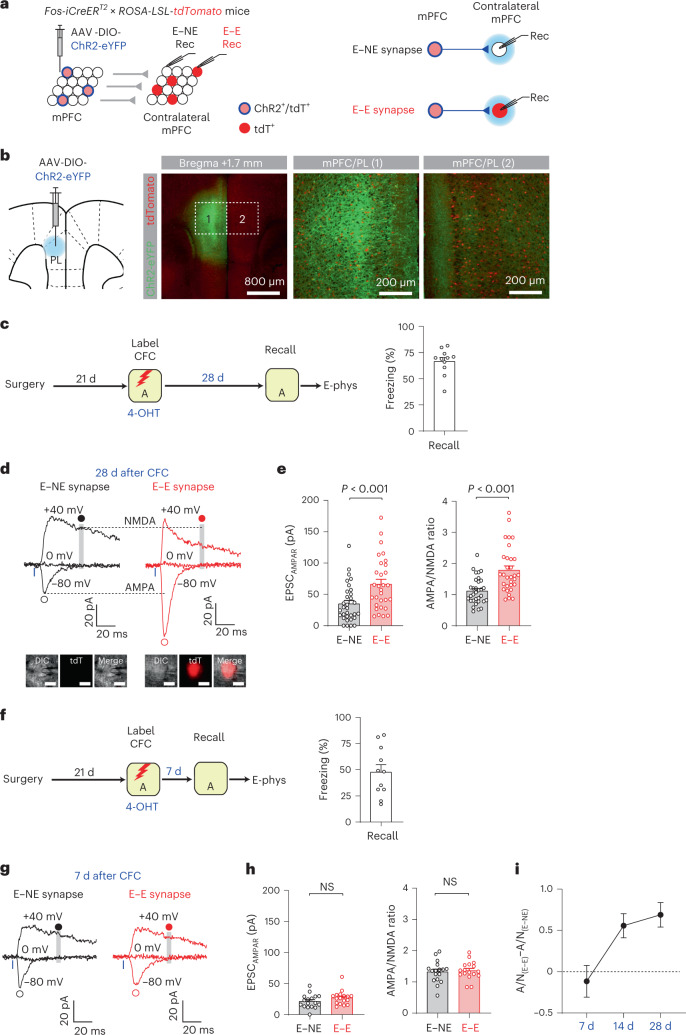
Progressive strengthening of interhemispheric excitatory connections between mPFC engram neurons during remote memory consolidation. **a**, Photostimulation activated ChR2^+^ engram inputs. Postsynaptic responses recorded in tdT^−^ nonengram (E–NE synapses) and tdT^+^ engram neurons (E–E synapses). **b**, Engram neurons in the AAV-injected mPFC (1) expressed ChR2-eYFP (green) and tdT (red). ChR2-eYFP^+^ axons and tdT^+^ engram neurons were detected in the contralateral mPFC (2). **c**, Experimental setup for **d** and **e**. Four weeks after CFC, mice were tested for remote fear memory recall (11 mice) and recording experiments were performed. **d**, Traces of EPSCs in E–NE (black) and E–E synapses (red). Blue light (blue bars) activated ChR2^+^ engram inputs and induced EPSCs recorded in a tdT^−^ nonengram neuron and an adjacent tdT^+^ engram neuron (red, inset; scale bar, 10 μm). EPSCs were recorded at –80, 0, and +40 mV in voltage-clamp mode in the presence of SR-95531. AMPAR EPSCs were recorded at –80 mV (open circles). NMDAR EPSCs were recorded at +40 mV (gray vertical lines and closed circles). **e**, Left: comparison of AMPAR EPSC (EPSC_AMPAR_) induced by the photostimulation of the same intensity (20.5 mW mm^–^^2^). Right: comparison of the AMPA/NMDA ratios. *n* = 32 (E–NE) and 31 (E–E). Two-way ANOVA with post hoc comparisons was used to analyze combined data in **e** and **h**. **f**, Experimental setup for **g** and **h**. Seven days after CFC, mice were tested for fear memory recall (11 mice), and recording experiments were performed. **g**, Traces of EPSCs in E–NE (black) and E–E synapses (red) induced and recorded as in **d**. **h**, Comparison of EPSC_AMPAR_ (left) and the AMPA/NMDA (right) ratios. *n* = 17 neurons/group. **i**, Comparison of difference in the AMPA/NMDA (A/N) ratio between E–NE and E–E synapses in mice examined 7 d (10 pairs), 14 d (15 pairs) and 28 d (30 pairs) after CFC. Data are presented as the mean ± s.e.m. Details of the statistical analyses are presented in Supplementary Table 2. Source data

**Extended Data Fig. 2:**
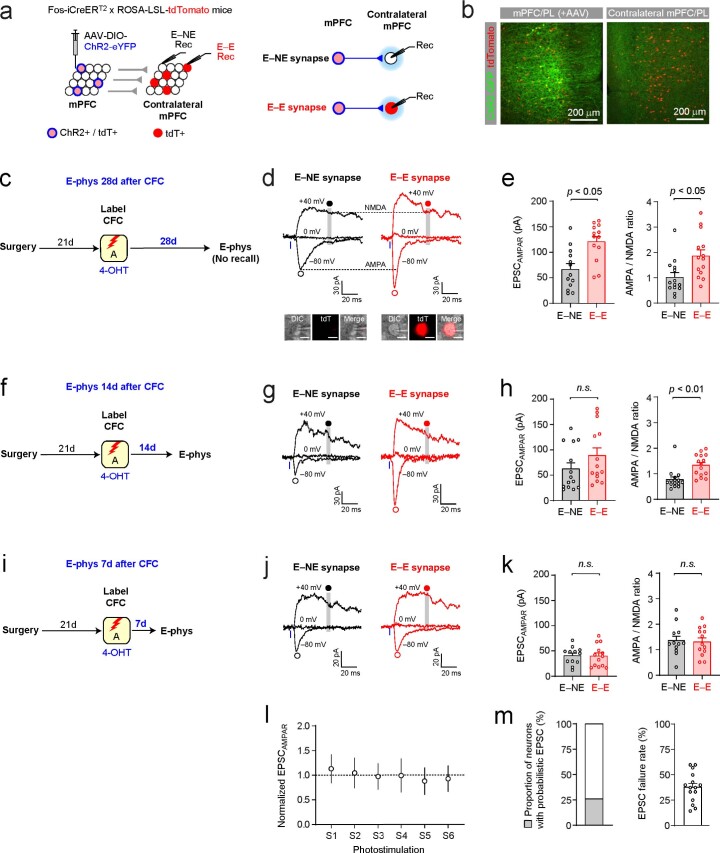
Progressive strengthening of interhemispheric excitatory connections between mPFC engram neurons during remote memory consolidation. (**a**) Photostimulation activated ChR2+ engram inputs. EPSCs were recorded in tdT− nonengram (E−NE synapses) and tdT+ engram neurons (E−E synapses) in contralateral mPFC. (**b**) *Left*: ChR2-eYFP+ (green) and tdT+ (red) engram neurons in AAV-injected mPFC. *Right*: ChR2-eYFP+ axons and tdT+ engram neurons in contralateral mPFC. (**c**) Experimental setup for (d, e). Four weeks after CFC, electrophysiological experiments (E-phys) were performed without memory recall (4 mice). (**d**) Traces of EPSCs in E−NE and E−E synapses. EPSCs were induced and recorded as in Fig. 2d. (**e**) *Left*: comparison of EPSC_AMPAR_ induced by 20.5 mW/mm^2^ photostimulation in tdT− and tdT+ neurons (13 pairs). *Right*: comparison of AMPA/NMDA ratios (14 pairs). Paired *t*-test. (**f**) Experimental setup for (g, h). Two weeks after CFC, recording experiments were performed (4 mice). (**g**) Traces of EPSCs in E−NE and E−E synapses. (**h**) Comparison of EPSC_AMPAR_ (14 pairs of tdT− and tdT+ neurons) and AMPA/NMDA ratios (15 pairs). Paired *t*-test. (**i**) Experimental setup for (j)-(k). Seven days after CFC, recording experiments were performed without memory recall (4 mice). (**j**) Traces of EPSCs in E−NE and E−E synapses. (**k**) Comparison of EPSC_AMPAR_ (12 pairs of tdT− and tdT+ neurons) and AMPA/NMDA ratios (12 pairs). Paired *t*-test. (**l**) Quantification of peak amplitudes of EPSC_AMPAR_ induced by 6 photostimulations (S1-S6, 20 s interval) and normalized to average peak amplitude in each neuron (57 neurons, data from (i–k) and Fig. 2f–h). (**m**) In 26.3% of 57 neurons examined in (l), photostimulation did not induce EPSC at least once (left), and EPSCs were probabilistic with an average failure rate of 37.8 ± 4.8%. Data are presented as the mean ± SEM in (e), (h), (k), and (m) or as the mean ± standard deviation in (l). Source data

**Extended Data Fig. 3:**
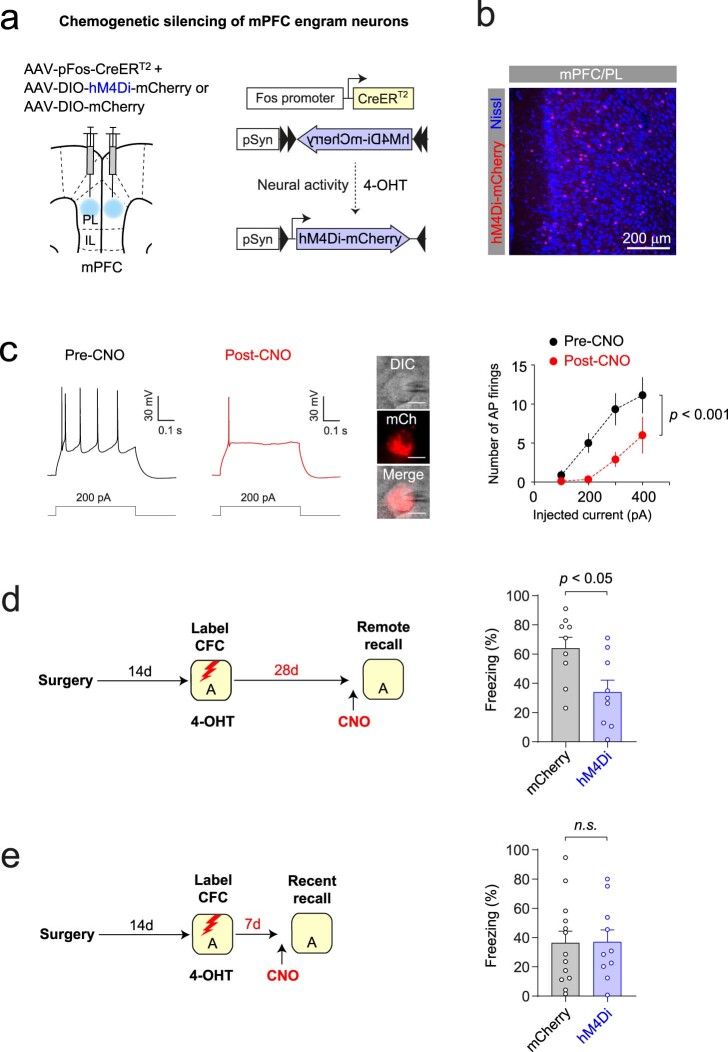
Chemogenetic silencing of mPFC engram neurons inhibited remote but not recent memory recall. (**a**) Experimental setup. A mixture of AAV-pFos-CreER^T2^ and AAV-DIO- hM_4_D_i_-mCherry (hM_4_D_i_ group) or AAV-DIO-mCherry (mCherry group) was bilaterally injected to the mPFC. mPFC engram neurons active during CFC expressed hM_4_D_i_-mCherry or mCherry. (**b**) Image showing mPFC engram neurons expressing hM_4_D_i_-mCherry (red). Blue fluorescence indicates Nissl stain. (**c**) *Left*: traces of AP firing before (pre-CNO) and 5 minutes after CNO application (10 μM, post-CNO). AP firing was induced by depolarizing current injection (500 ms long) and recorded in the same hM4Di-mCherry (mCh)-expressing mPFC neuron (inset; scale bar, 10 μm) in current-clamp mode. *Right*: summary plot of AP firing in 9 mPFC neurons expressing hM_4_D_i_-mCherry. *** *p* < 0.001 (pre-CNO versus post-CNO, repeated measures two-way ANOVA). (**d**) *Left*: mPFC engram neurons active during CFC in Context A were labeled with hM_4_D_i_-mCherry or mCherry. Four weeks after CFC, the mice received a CNO injection and were tested for fear memory recall in the same context 45–60 minutes later. *Right*: summary plot showing the average freezing time in the hM_4_D_i_ (9 mice) and mCherry groups (9 mice). Unpaired *t*-test. (**e**)*Left*: mPFC engram neurons active during CFC in Context A were labeled with hM_4_D_i_-mCherry or mCherry. Seven days after CFC, the mice received a CNO injection and were tested for fear memory recall in the same context 45–60 minutes later. *Right*: summary plot showing the average freezing time in the hM_4_D_i_ (10 mice) and mCherry groups (13 mice). Unpaired *t*-test. Data are presented as the mean ± SEM. Source data

We next examined if remote memory consolidation might strengthen connections between mPFC engram neurons to store remote contextual memories. AAV-DIO-ChR2-eYFP was unilaterally injected into the mPFC/PL in *Fos-iCreER*^*T2*^ × *ROSA-LSL-tdTomato* mice (Fig. 2a,b). Four weeks after CFC, the mice were tested for remote memory recall and brain slices were prepared for electrophysiological recordings (Fig. 2c). Engram neurons in the AAV-injected mPFC expressed ChR2-eYFP and tdT, whereas engram neurons in the contralateral mPFC expressed only tdT (Fig. 2b). ChR2-eYFP^+^ axons of mPFC engram neurons, which we termed ‘mPFC engram inputs’, were sparsely distributed in the contralateral mPFC (Fig. 2b). Photostimulation of ChR2^+^ interhemispheric mPFC engram inputs induced excitatory postsynaptic currents (EPSCs) recorded in mPFC layer 2/3 pyramidal neurons using the whole-cell patch-clamp technique. EPSCs recorded in tdT^+^ engram neurons and tdT^-^ nonengram (NE) neurons reflected synaptic responses in engram inputs to engram neurons (E–E synapses) and those inputs to nonengram neurons (E–NE synapses), respectively (Fig. 2a). Compared with E–NE synapses, E–E synapses displayed larger AMPA receptor (AMPAR)-mediated EPSCs (Fig. 2d,e). To compare synaptic strength, we recorded both AMPAR and NMDA receptor (NMDAR)-mediated EPSCs in the same mPFC neurons and calculated the AMPA/NMDA ratio^17,18^, which was also higher in E–E synapses than in NE–E synapses (Fig. 2d,e). Thus, interhemispheric mPFC engram inputs were more strongly connected to mPFC engram neurons than to nonengram neurons 28 d after CFC. Such synaptic changes were also observed 14 d but not 7 d after CFC (Fig. 2f–i and Extended Data Fig. 2f–h), suggesting that mPFC E–E synapses were gradually strengthened during systems consolidation. The strengthening of mPFC E–E synapses was not induced by remote memory recall because the same temporal pattern of synaptic changes was observed without memory recall before recordings (Extended Data Fig. 2a–e,i–m). Consistent with this, silencing of mPFC engram neurons inhibited memory recall 28 d but not 7 d after CFC (Extended Data Fig. 3), suggesting that remote memory recall requires the activity of mPFC engram neurons.

**Extended Data Fig. 4:**
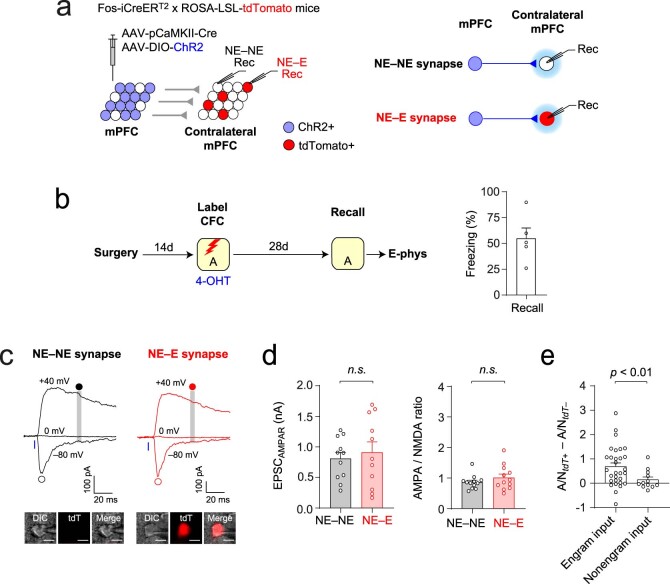
mPFC nonengram inputs to mPFC engram neurons were not strengthened after remote memory consolidation. (**a**) *Left*: AAV-pCaMKII-Cre and AAV-DIO-ChR2-eYFP were unilaterally injected into the mPFC/PL in Fos-iCreER^T2^ × ROSA-LSL-tdTomato mice. ChR2-eYFP was globally expressed in CaMKII+ pyramidal neurons of the AAV-injected mPFC (blue), whereas mPFC engram neurons expressed tdT (red). *Right*: in the contralateral mPFC, photostimulation activated ChR2+ axons of nonengram mPFC neurons. Postsynaptic responses recorded in nonengram (NE, tdT−) and engram neurons (E, tdT+) reflected those in nonengram inputs to nonengram neurons (NE−NE synapses) and to engram neurons (NE−E synapses), respectively. (**b**) *Left*: mice were fear conditioned in Context A and received a 4-OHT injection to label mPFC engram neurons with tdT. They were tested for contextual memory recall 28 days later. *Right*: quantification of freezing behavior during memory recall (5 mice). (**c**) Representative traces of EPSCs in NE−NE (black) and NE−E synapses (red). EPSCs were induced by the photostimulation (blue bars) of nonengram mPFC inputs and recorded in a pair of tdT− nonengram (NE−NE synapses) and tdT+ engram neurons (NE−E synapses). AMPAR and NMDAR EPSCs were recorded and quantified as in Fig. 2d. Scale bar, 10 μm (inset). (**d**) *Left*: comparison of the peak amplitude of AMPAR EPSC (EPSC_AMPAR_) induced by photostimulation of the same intensity (20.5 mW/mm^2^) and recorded in 11 pairs of nonengram (NE−NE synapses) versus engram neurons (NE−E synapses). *Right*: comparison of AMPA/NMDA ratios in 12 pairs of nonengram (NE−NE synapses) versus engram neurons (NE−E synapses). *n.s*., nonsignificant. Paired *t*-test. (**e**) Comparison of difference in AMPA/NMDA (A/N) ratio between E−NE and E−E synapses (engram inputs; 30 pairs, data from Fig. 2e) with difference in AMPA/NMDA ratio between NE−NE and NE−E synapses (nonengram inputs; 12 pairs, data from (d)). Unpaired *t*-test. Data are presented as the mean ± SEM. Source data

We also examined whether synaptic strengthening associated with systems consolidation was input specific. ChR2-eYFP was globally expressed in CaMKII^+^ pyramidal neurons in the AAV-injected mPFC (Extended Data Fig. 4a,b). As engram neurons constitute only a small subset of CaMKII^+^ mPFC neurons (Extended Data Fig. 1e,f), most ChR2-eYFP^+^ axons were nonengram inputs. Four weeks after CFC, photostimulation of ChR2-eYFP^+^ axons induced EPSCs in tdT^+^ engram (NE–E synapses) and tdT^−^ nonengram neurons (NE–NE synapses) in the contralateral mPFC. AMPAR-mediated EPSCs or the AMPA/NMDA ratio did not differ between NE–E and NE–NE synapses (Extended Data Fig. 4c–e), suggesting that nonengram inputs to mPFC engram neurons were not strengthened. Thus, remote memory consolidation involves synapse-specific strengthening of excitatory connections between mPFC engram neurons, which we termed the ‘mPFC engram circuit’.

**Extended Data Fig. 5:**
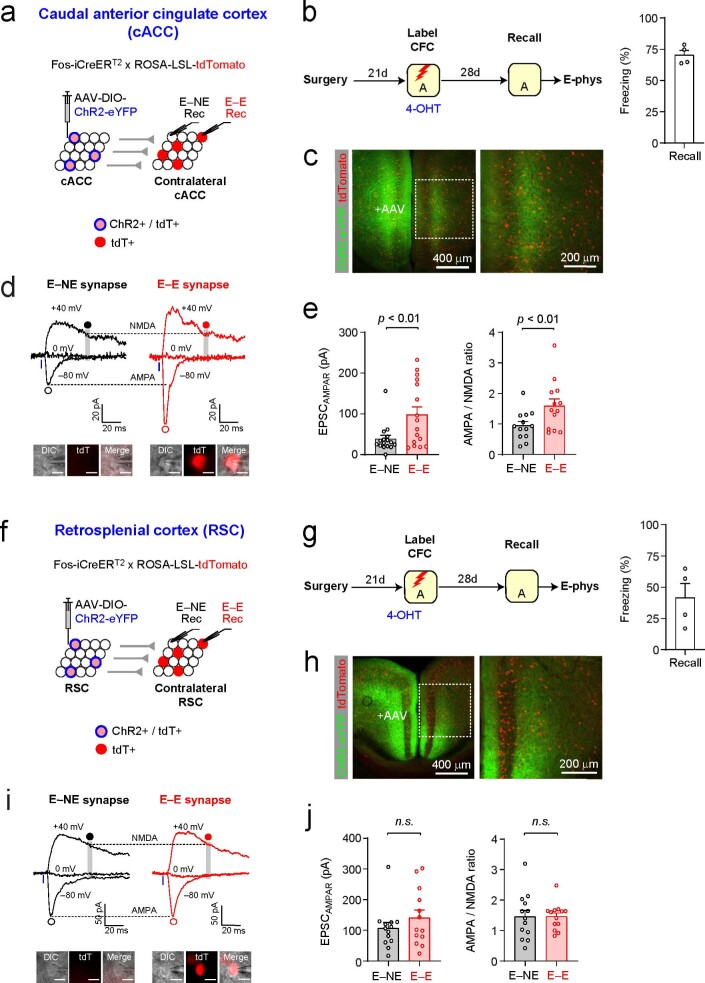
Remote memory consolidation strengthened interhemispheric excitatory connections between engram neurons in the cACC, but not in the RSC. (**a**) Experimental setup for (b–e). AAV-DIO-ChR2-eYFP was unilaterally injected into caudal ACC (cACC) in Fos-iCreER^T2^ × ROSA-LSL-tdTomato mice. Photostimulation of ChR2+ engram inputs induced postsynaptic responses in nonengram (tdT−, E−NE synapses) and engram neurons (tdT+, E−E synapses) in contralateral cACC. (**b**) Mice were tested for memory recall 28 days after CFC (4 mice). (**c**) Engram neurons expressed ChR2-eYFP (green) and tdT (red) in AAV-injected cACC (left). Contralateral cACC indicated by a dotted square is magnified in the right panel, showing tdT+ engram neurons and ChR2-eYFP+ axons. (**d**)Traces of EPSCs induced and recorded as in Fig. 2d. Scale bar, 10 μm (inset). (**e**) *Left*: comparison of the amplitude of AMPAR EPSC induced by 20.5 mW/mm^2^ photostimulation and recorded in tdT− and tdT+ neurons (16 pairs). *Right*: comparison of AMPA/NMDA ratio (13 pairs). Paired *t*-test. (**f**) Experimental setup for (g–j). AAV-DIO-ChR2-eYFP was unilaterally injected into retrosplenial cortex (RSC) in Fos-iCreER^T2^ × ROSA-LSL-tdTomato mice. Photostimulation of ChR2+ engram inputs induced postsynaptic responses in nonengram (tdT−, E−NE synapses) and engram neurons (tdT+, E−E synapses) in contralateral RSC. (**g**) Mice were tested for memory recall 28 days after CFC (4 mice). (**h**) Engram neurons expressed ChR2-eYFP (green) and tdT (red) in AAV-injected RSC (left). Contralateral RSC indicated by a dotted square is magnified in the right panel, showing tdT+ engram neurons and ChR2-eYFP+ axons. (**i**) Traces of EPSCs induced and recorded as in Fig. 2d. Scale bar, 10 μm (inset). (**j**) *Left*: comparison of the amplitude of AMPAR EPSC induced by 20.5 mW/mm^2^ photostimulation and recorded in tdT− and tdT+ neurons (13 pairs). *Right*: comparison of AMPA/NMDA ratio (13 pairs). Paired *t*-test. Data are presented as the mean ± SEM. Source data

We next examined whether systems consolidation also strengthened engram circuits in the caudal ACC (cACC) and retrosplenial cortex (RSC) implicated in the consolidation and retrieval of remote contextual memories^9,19,20^ (Extended Data Fig. 5). In the cACC, both AMPAR EPSCs and the AMPA/NMDA ratio were substantially larger in E–E synapses than in E–NE synapses 28 d after CFC, whereas such differences were not detected in the RSC. These results suggest that certain neocortical engram circuits are more likely to undergo synaptic changes during systems consolidation.

### CREB-dependent strengthening of mPFC engram circuit

**Fig. 3:**
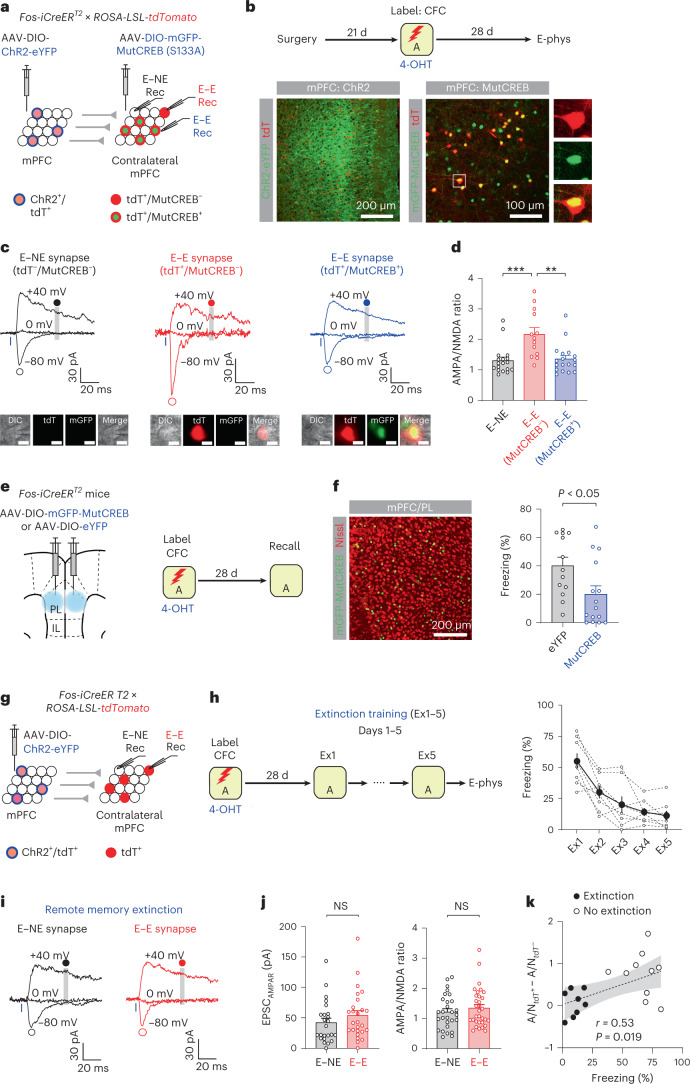
Strengthening of mPFC engram circuit required CREB and remote memory extinction weakened mPFC engram circuit. **a**, Experimental setup for **b**–**d**. Photostimulation of ChR2^+^ engram inputs induced EPSCs in nonengram (tdT^−^/MutCREB^−^, E–NE synapses) and engram neurons (tdT^+^/MutCREB^−^ or tdT^+^/MutCREB^+^, E–E synapses). **b**, Top: 28 d after CFC, recording experiments were performed. Bottom: images showing ChR2-eYFP^+^ (green) and/or tdT^+^ (red) mPFC engram neurons (left) and those labeled with mGFP-MutCREB (green) and/or tdT^+^ (red) in contralateral mPFC (right). **c**, Traces of EPSCs in E–NE (tdT^−^/MutCREB^−^, black), E–E (tdT^+^/MutCREB^−^, red) and E–E synapses (tdT^+^/MutCREB^+^, blue). AMPAR EPSCs and NMDAR EPSCs were recorded as in Fig. 2d. Scale bar, 10 μm (inset). **d**, Comparison of AMPA/NMDA ratios between E–NE (18 cells), E–E (tdT^+^/MutCREB^−^, 13 cells) and E–E synapses (tdT^+^/MutCREB^+^, 18 cells). One-way ANOVA with post hoc comparisons (**P < 0.01, ***P < 0.001). **e**, Experimental setup for **f**. mPFC engram neurons expressed mGFP-MutCREB or eYFP. Mice were tested for memory recall 28 d after CFC. **f**, Left: image showing mGFP-MutCREB^+^ mPFC neurons (green). Right: freezing behavior during memory recall in MutCREB (15 mice) and eYFP groups (13 mice). Unpaired *t*-test. **g**, Experimental setup for **h**–**j**. Photostimulation of mPFC engram inputs induced EPSCs in tdT^−^ nonengram (E–NE synapses) and tdT^+^ engram neurons (E–E synapses). **h**, Left: 28 d after CFC, mice received extinction training for 5 d (Ex1-5). Right: freezing behavior during extinction training (eight mice). **i**, Traces of EPSCs in E–NE (black) and E–E synapses (red). **j**, Comparison of EPSC_AMPAR_ (24 cells for E–NE, 26 cells for E–E synapses) and AMPA/NMDA ratio (28 cells for E–NE, 32 cells for E–E synapses). Unpaired *t-*test. **k**, Average difference in AMPA/NMDA ratio between tdT^+^ and tdT^–^ neurons in each mouse positively correlated with freezing behavior (Pearson correlation test). In the extinction group (eight mice), freezing scores during the last extinction session were used. In the no extinction group (11 mice), data in Fig. 2c–e were used. Gray shaded area indicates 95% confidence bands on the best-fitting regression line. Data are presented as the mean ± s.e.m. Details of the statistical analyses are presented in Supplementary Tables 1 and 2. Source data

In a previous study, the inhibition of CREB in mPFC engram neurons prevented remote memory consolidation^10^. Since CREB is essential for neuronal plasticity^21^, CREB inhibition may block the strengthening of mPFC engram circuit, thereby preventing systems consolidation. To test this, we inhibited CREB in mPFC engram neurons with dominant-negative mutant CREB(S133A) or MutCREB^10^ after CFC and examined how this affected the strengthening of mPFC engram circuit. mPFC engram neurons expressed ChR2-eYFP and tdT, while some tdT^+^ engram neurons in the contralateral mPFC expressed mGFP-MutCREB (Fig. 3a,b). Photostimulation of ChR2^+^ engram inputs induced EPSCs in tdT^+^/MutCREB^−^ engram neurons (E–E synapses), tdT^+^/MutCREB^+^ engram neurons (E–E synapses) and tdT^−^/MutCREB^−^ nonengram neurons (E–NE synapses) (Fig. 3c). The AMPA/NMDA ratio was substantially larger in tdT^+^/MutCREB^−^ engram neurons than in tdT^+^/MutCREB^+^ engram neurons or tdT^−^/MutCREB^−^ nonengram neurons and did not differ between tdT^+^/MutCREB^+^ neurons and tdT^−^/MutCREB^−^ neurons (Fig. 3c,d), indicating that MutCREB expression in postsynaptic mPFC neurons prevented the strengthening of mPFC engram circuit. Thus, CREB in mPFC engram neurons is indispensable for the strengthening of the mPFC circuit during systems consolidation. Moreover, overexpression of MutCREB in mPFC engram neurons after CFC also inhibited remote memory recall (Fig. 3e,f), suggesting the critical role of CREB in remote memory consolidation and/or recall.

### Extinction of remote memories weakened mPFC engram circuit

After CFC, repeated exposure to the threat-predictive context without the US induces the extinction of contextual fear memories. We examined how remote memory extinction affected mPFC engram circuit. Four weeks after CFC, extinction training for 5 d gradually decreased freezing behavior in Context A (Fig. 3g,h). After extinction training, neither AMPAR EPSCs nor the AMPA/NMDA ratio differed between E–E and E–NE synapses in the mPFC (Fig. 3i,j), suggesting that remote memory extinction weakened E–E synapses previously strengthened during systems consolidation. Overall, the average difference in the AMPA/NMDA ratio between E–E and E–NE synapses in each mouse correlated with freezing behavior during the last recall session (Fig. 3k), suggesting that systems consolidation strengthens mPFC engram circuit, while remote memory extinction weakens the engram circuit.

### Strengthening of local mPFC circuit in systems consolidation

**Fig. 4:**
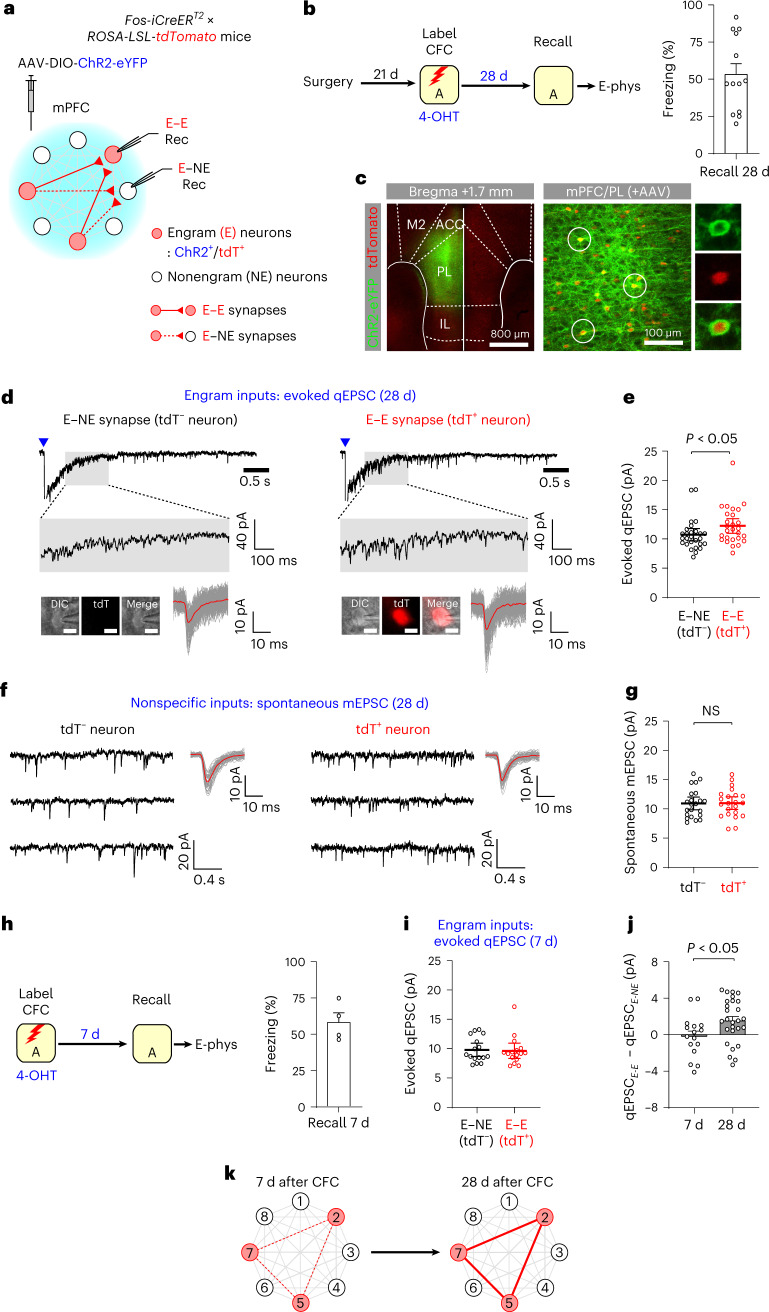
Progressive strengthening of local recurrent excitatory connections between mPFC engram neurons during remote memory consolidation. **a**, Photostimulation activated local recurrent axons of ChR2^+^ engram neurons and induced postsynaptic responses recorded in engram (tdT^+^, E–E synapses) and nonengram neurons (tdT^−^, E–NE synapses). **b**, Experimental setup for **c**–**g**. Four weeks after CFC, the mice were tested for remote memory recall (13 mice). **c**, Images showing ChR2-eYFP^+^ (green) and tdT^+^ (red) mPFC engram neurons. **d**, Traces of evoked qEPSCs induced by the photostimulation (blue triangles) of local recurrent engram inputs and recorded in tdT^−^ nonengram (E–NE synapses) and tdT^+^ engram neurons (E–E synapses). The average qEPSC (red) was overlaid onto individual qEPSCs (gray). Scale bar, 10 μm (inset). **e**, Comparison of the average peak amplitude of evoked qEPSCs recorded in 27 pairs of nonengram (E–NE synapses) and engram neurons (E–E synapses). Two-way ANOVA with post hoc comparisons was used to analyze combined data in **e** and **g**. **f**, Traces of spontaneous mEPSCs in nonspecific inputs to tdT^–^ nonengram and tdT^+^ engram neuron. mEPSCs were recorded at −80 mV in the presence of TTX. Average mEPSCs (red) were overlaid onto individual mEPSCs (gray). **g**, Comparison of the peak amplitude of spontaneous mEPSCs recorded in 22 pairs of nonengram and engram neurons. **h**, Experimental setup for **i**. Seven days after CFC, the mice were tested for memory recall (four mice). **i**, Comparison of the peak amplitude of evoked qEPSCs recorded in 16 pairs of nonengram (E–NE synapses) and engram neurons (E–E synapses) 7 d after CFC. **j**, Comparison of difference in evoked qEPSC amplitude between E–NE and E–E synapses in mice examined 7 d (16 pairs) versus 28 d after CFC (27 pairs). Unpaired *t*-test. **k**, Local recurrent excitatory connections between mPFC engram neurons were gradually strengthened during systems consolidation. Data are presented as the mean ± s.e.m. in **b**, **h** and **j**, whereas data are presented as the mean ± 95% confidence interval in **e**, **g** and **i**. Details of the statistical analyses are presented in Supplementary Tables 1 and 2. Source data

Our results suggest that remote memory consolidation involves the strengthening of interhemispheric connections between mPFC engram neurons. We next examined whether *local* excitatory connections between mPFC engram neurons were also strengthened after systems consolidation. Four weeks after CFC, mPFC engram neurons expressed ChR2 and tdT (Fig. 4a–c). Photostimulation of ChR2^+^ engram neurons induced EPSCs recorded in adjacent engram and nonengram neurons in the ipsilateral mPFC. In engram neurons, we isolate EPSCs from ChR2-mediated photocurrents by inducing asynchronous glutamate release from presynaptic engram inputs in Ca^2+^-free and 4 mM Sr^2+^-containing extracellular solution^22^, which also contained tetrodotoxin (TTX, 1 μM) and 4-aminopyridine (4-AP, 1 mM) to prevent postsynaptic EPSCs^23^ (Fig. 4d). Evoked quantal EPSCs (qEPSCs) were recorded in tdT^+^ engram and tdT^−^ nonengram neurons at −80 mV in voltage-clamp mode and calculated the average peak amplitude of all detected qEPSCs in each neuron as an index of synaptic strength^24^. qEPSCs amplitude was substantially larger in engram neurons than in nonengram neurons (Fig. 4e), indicating stronger local excitatory connections in E–E synapses than in E–NE synapses 28 d after CFC. To compare synaptic strength in nonspecific inputs to mPFC engram versus nonengram neurons, we also recorded spontaneous miniature EPSCs (mEPSCs), whose amplitude did not differ between engram and nonengram neurons (Fig. 4f,g), suggesting that systems consolidation did not alter synaptic strength in nonspecific inputs to engram neurons.

**Extended Data Fig. 6:**
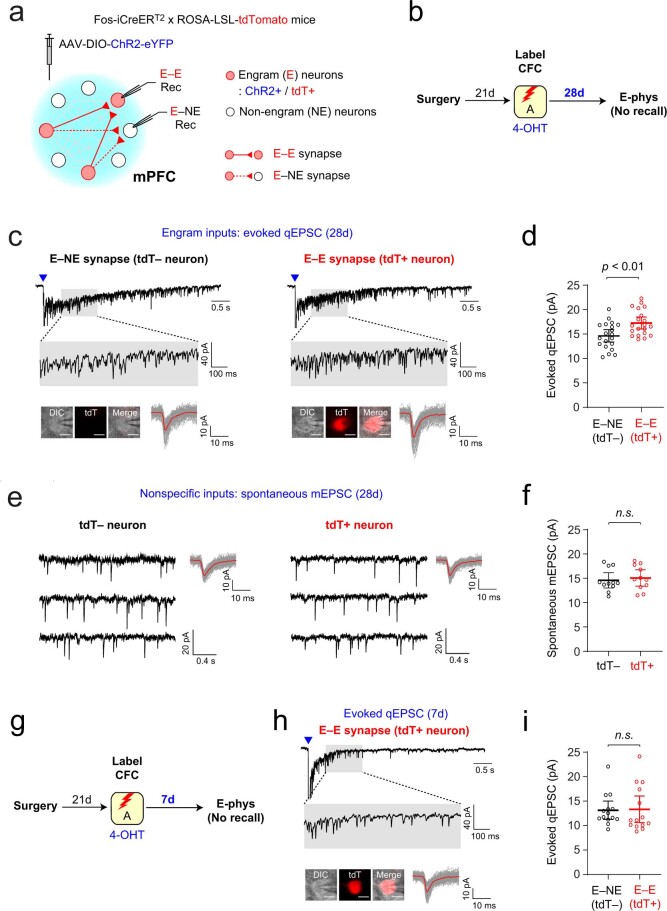
Strengthening of local recurrent excitatory connections between mPFC engram neurons during remote memory consolidation. (**a**) Photostimulation activated local recurrent axons of ChR2+ engram neurons and induced postsynaptic responses recorded in engram (tdT+, E−E synapses) and nonengram neurons (tdT−, E−NE synapses). (**b**) Experimental setup for (c–f). Four weeks after CFC, electrophysiological experiments (E-phys) were performed without memory recall (5 mice). (**c**) Traces of evoked qEPSCs induced by photostimulation (blue triangles) of local recurrent engram inputs and recorded in tdT– nonengram (E−NE synapses) and tdT+ engram neurons (E−E synapses) as in Fig. 4d. Average qEPSC (red) was overlaid onto individual qEPSCs (gray). Scale bar, 10 μm (inset). (**d**) Comparison of peak amplitude of evoked qEPSCs recorded 20 pairs of tdT– nonengram (E−NE synapses) and tdT+ engram neurons (E−E synapses). Paired *t*-test. (**e**) Traces of spontaneous mEPSCs in nonspecific inputs to tdT– nonengram and tdT+ engram neuron. mEPSCs were recorded at −80 mV in the presence of TTX as in Fig. 4f. Average mEPSCs (red) was overlaid onto individual mEPSCs (gray). (**f**) Comparison of peak amplitude of spontaneous mEPSCs recorded in 11 pairs of tdT– nonengram and tdT+ engram neurons. Paired *t*-test. (**g**) Experimental setup for (h, i). Seven days after CFC, electrophysiological experiments (E-phys) were performed without memory recall (4 mice). (**h**) Traces of evoked qEPSCs induced by photostimulation (blue triangles) of local recurrent engram inputs and recorded in a tdT+ engram neurons (E−E synapses) as in Fig. 4d. Average qEPSC (red) was overlaid onto individual qEPSCs (gray). Scale bar, 10 μm (inset). (**i**) Comparison of peak amplitude of evoked qEPSCs recorded qEPSCs recorded in 14 pairs of tdT– nonengram (E−NE synapses) and tdT+ engram neurons (E−E synapses) 7 days after CFC. Paired *t*-test. Data are presented as the mean ± 95% confidence interval. Source data

**Extended Data Fig. 7:**
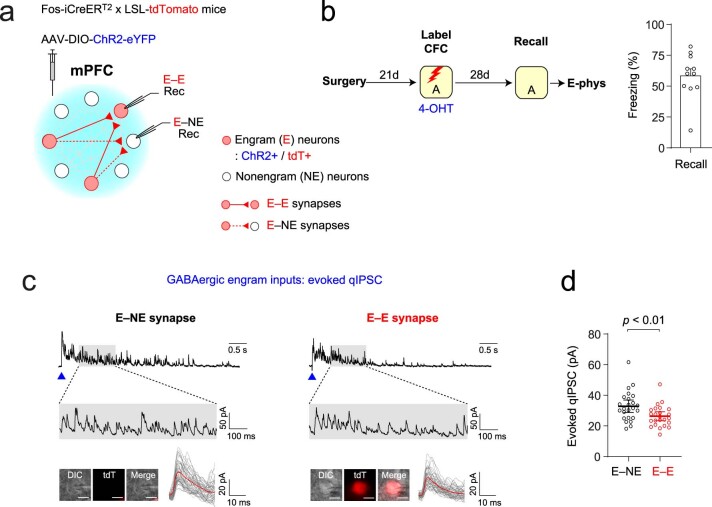
Comparison of synaptic strength in local inhibitory engram inputs to mPFC engram versus nonengram pyramidal neurons. (**a**) *Top*: AAV-DIO-ChR2-eYFP was injected into the mPFC/PL in Fos-iCreER^T2^ × ROSA-LSL-tdTomato mice. mPFC engram neurons expressed ChR2-eYFP and tdT. Photostimulation activated axons of local GABAergic engram neurons and induced inhibitory postsynaptic responses recorded in engram (E−E synapses) and nonengram pyramidal neurons (E−NE synapses). (**b**) *Left*: 28 days after CFC, the mice were tested for the recall of remote contextual fear memories, and electrophysiological experiments were performed. *Right*: quantification of freezing behavior during memory recall (11 mice). Data are presented as the mean ± SEM. (**c**) Representative traces of quantal IPSCs (qIPSCs) induced by the photostimulation of local GABAergic engram inputs and recorded in a tdT− nonengram pyramidal neuron (E−NE synapses) and a tdT+ engram pyramidal neuron (E−E synapses) in the mPFC. Photostimulation (blue triangles) activated local GABAergic inputs of ChR2+ engram neurons and induced IPSCs in postsynaptic pyramidal neurons. qIPSCs were recorded at 0 mV in voltage-clamp mode. Presynaptic GABA release was desynchronized in Ca^2+^-free and 4 mM Sr^2+^-containing extracellular solution. In each neuron, peak amplitudes of well-separated qIPSCs recorded 0.5–1.5 s after photostimulation (gray areas) were calculated and averaged. The average qIPSC (red) was overlaid onto individual qIPSC traces (gray). TTX (1 μM) and 4-AP (1 mM) were added in the extracellular solution to block polysynaptic IPSCs. NBQX (10 μM) was also added to block excitatory glutamatergic transmission. Scale bar, 10 μm (inset). (**d**) Comparison of the peak amplitude of evoked qIPSCs in GABAergic engram inputs to 24 pairs of nonengram neurons (E−NE synapses) versus engram neurons (E−E synapses). Paired *t*-test. Data are presented as the mean ± 95% confidence interval. Source data

We also recorded evoked qEPSC 7 d after CFC and found no difference in qEPSC amplitude between E–E and E–NE synapses (Fig. 4h–j), indicating that local mPFC engram circuit was not yet strengthened 7 d after CFC (Fig. 4k). The strengthening of local mPFC engram circuit was not induced by remote memory recall as the same temporal pattern of synapse-specific strengthening was observed without memory recall before recordings (Extended Data Fig. 6). As mPFC GABAergic interneurons are involved in encoding conditioned fear memories^25,26^, we next examined synaptic changes in inhibitory engram inputs to mPFC pyramidal neurons. CFC induced ChR2-eYFP and tdT expression in both excitatory and inhibitory engram neurons (Extended Data Fig. 7a,b). After 28 d, we photostimulated ChR2^+^ mPFC engram inputs and recorded inhibitory postsynaptic currents (IPSCs) in tdT^−^ or tdT^+^ pyramidal neurons at 0 mV in the voltage-clamp mode in the presence of Sr^2+^, TTX, 4-AP and NBQX. qIPSC amplitude was substantially smaller in tdT^+^ neurons than in tdT^−^ neurons (Extended Data Fig. 7c,d), indicating weaker GABAergic engram inputs to mPFC engram neurons than those inputs to nonengram neurons. Thus, mPFC engram neurons receive weaker inhibitory engram inputs than other neurons.

### Maturation of mPFC engram requires hippocampal dentate gyrus activity

**Fig. 5:**
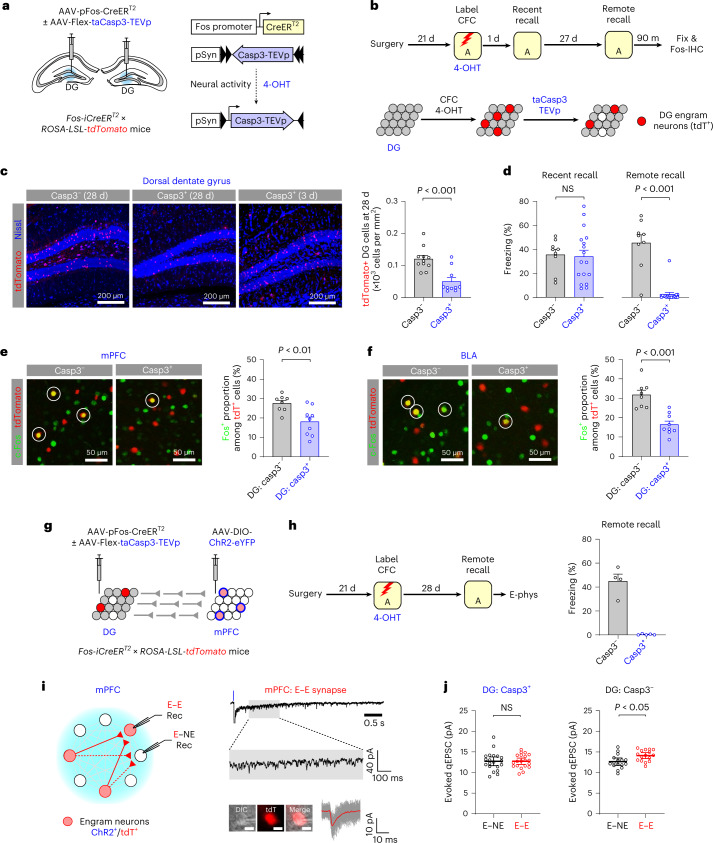
Ablation of DG engram neurons after learning inhibited the reactivation of mPFC engram neurons during memory recall and the strengthening of mPFC engram circuit. **a**, Experimental setup for **b**–**f**. **b**, Top: mice were tested for memory recall 1 and 28 d after CFC. Brain tissues were immunolabeled for c-Fos (Fos-IHC) after remote memory recall. Bottom: in Casp3^+^ group, DG engram neurons expressed tdT and taCasp3-TEVp (red circles), resulting in cell death (open circles). **c**, Left: Casp3-mediated cell death resulted in lower tdT^+^ DG cell density in Casp3^+^ group than in Casp3^−^ group 28 d but not 3 d after CFC. Right: tdT^+^ DG cell density in Casp3^+^/28 d (10 mice) versus Casp3^−^/28 d groups (11 mice). Unpaired *t*-test. **d**, Freezing behavior during memory recall in Casp3^+^ (17 mice) and Casp3^−^ groups (nine mice). Repeated measures ANOVA with post hoc comparisons. **e**, Left: images showing tdT^+^ and/or c-Fos^+^ mPFC/PL neurons. Both tdT^+^ and c-Fos^+^ neurons are circled. Right: c-Fos^+^ proportion among all tdT^+^ mPFC/PL neurons in Casp3^−^ (eight mice) and Casp3^+^ groups (nine mice). Unpaired *t*-test. **f**, Left: images showing tdT^+^ and/or c-Fos^+^ BLA neurons. Right: c-Fos^+^ proportion among all tdT^+^ BLA neurons in Casp3^−^ (eight mice) and Casp3^+^ groups (nine mice). Unpaired *t*-test. **g**, Experimental setup for **h**–**j**. DG engram neurons underwent cell death in Casp3^+^ group. mPFC engram neurons expressed ChR2-eYFP and tdT. **h**, Mice in Casp3^+^ (five mice) and Casp3^−^ groups (four mice) were tested for memory recall 28 d after CFC. **i**, Left: photostimulation activated local recurrent axons of ChR2^+^ engram neurons and induced qEPSCs in nonengram (E–NE synapses) and tdT^+^ engram neurons (E–E synapses) as in Fig. 4d. Right: trace of evoked qEPSCs in E–E synapses. Scale bar, 10 μm (inset). **j**, Comparison of qEPSC amplitude between nonengram (E–NE synapses) and engram neurons (E–E synapses) in Casp3^+^ group (20 pairs, left) and in Casp3^−^ group (16 pairs, right). Paired *t*-test. Data are presented as the mean ± s.e.m. in **c**, **d**, **e**, **f** and **h** or as the mean ± 95% confidence interval in **j**. Details of statistical analyses are presented in Supplementary Tables 1 and 2. Source data

The dorsal dentate gyrus (DG) has a critical role in mPFC engram maturation during systems consolidation^12^. To determine how DG contributes to systems consolidation, we genetically ablated DG engram neurons active during CFC and examined how this affected the reactivation of mPFC engram during remote memory recall and the strengthening of mPFC engram circuit. In the Casp3^+^ group, AAV-Flex-taCasp3-TEVp and AAV-pFos-CreER^T2^ were bilaterally injected into the dorsal DG in *Fos-iCreER*^*T2*^ × *ROSA-LSL-tdTomato* mice (Fig. 5a). In this group, DG engram neurons expressed tdT and taCasp3-TEVp and underwent taCaspase-3-mediated cell death^27^, resulting in efficient ablation of DG engram neurons 28 d but not 3 d after CFC (Fig. 5b,c). One day after CFC, mice showed robust freezing behavior in Context A, while mice in the Casp3^+^ group displayed much less freezing behavior than mice in no Caspase3 (Casp3^−^) control group 28 d after CFC (Fig. 5d). This suggests that the ablation of DG engram neurons inhibited the consolidation and/or retrieval of remote contextual memories. In both groups, engram neurons were labeled with tdT, whereas neurons active during remote memory recall were immunostained for c-Fos. In both the mPFC/PL and BLA, the proportion of c-Fos^+^ neurons among all tdT^+^ engram neurons was substantially lower in the Casp3^+^ group than in the Casp3^−^ group (Fig. 5e,f), indicating reduced reactivation of engram neurons during remote memory recall when DG engram neurons were ablated.

**Extended Data Fig. 8:**
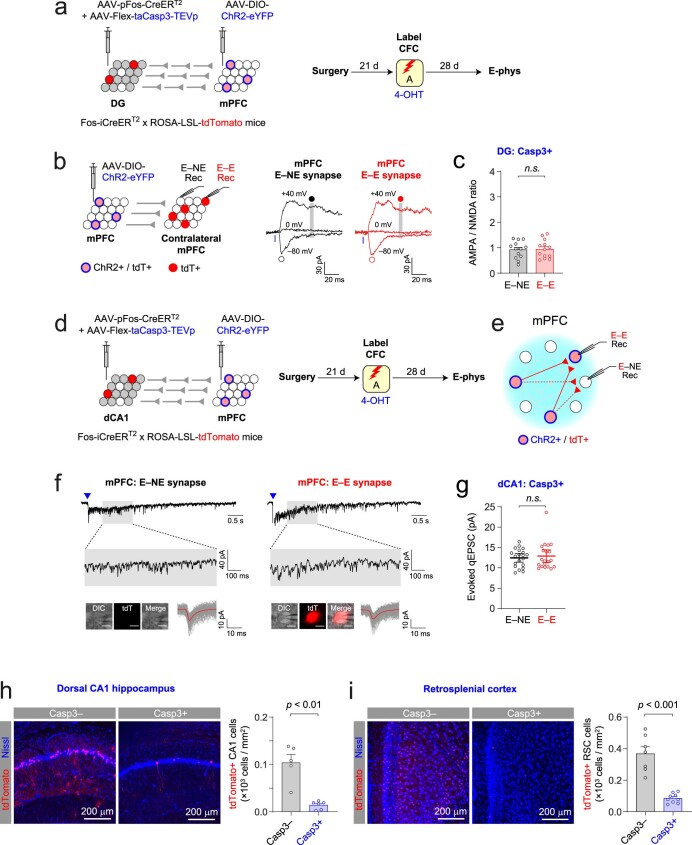
Ablation of dorsal DG or CA1 hippocampal engram neurons inhibited the strengthening of mPFC engram circuits. (**a**) Experimental setup for (b, c). DG engram neurons underwent Casp3-mediated cell death. Engram neurons in AAV-injected mPFC expressed ChR2-eYFP and tdTomato (tdT). Recordings experiments were performed 28 days after CFC (4 mice). (**b**) *Left*: photostimulation activated ChR2+ interhemispheric engram inputs and induced EPSCs in tdT− nonengram (E−NE) and tdT+ engram neurons (E−E). *Right*: traces of EPSCs recorded as in Fig. 2d in tdT− and tdT+ neurons. (**c**) Comparison of AMPA/NMDA ratios (right) in 13 pairs of mPFC nonengram (E−NE) and engram neurons (E−E). Paired *t*-test. (**d**) Experimental setup for (e–g). Engram neurons in dorsal CA1 underwent Casp3-mediated cell death. mPFC engram neurons expressed ChR2-eYFP and tdT. Recording experiments were performed 28 days after CFC (5 mice). (**e**) Photostimulation activated local recurrent axons of ChR2+ engram neurons and induced EPSCs in mPFC nonengram (E−NE) and tdT+ engram neurons (E−E). (**f**) Traces of evoked qEPSCs recorded as in Fig. 4d in E−NE and E−E synapses. Scale bar, 10 μm. (**g**) Comparison of peak amplitude of evoked qEPSCs recorded in 20 pairs of mPFC nonengram (E−NE) and engram neurons (E−E). Paired *t*-test. (**h**) AAVs were injected to dCA1 as in Fig. 6j in ROSA-LSL-tdTomato mice. *Left*: Casp3-mediated cell death in Casp3+ group resulted in lower tdT+ dCA1 cell density compared with Casp3− group. *Right*: tdT+ dCA1 cell density in Casp3+ (6 mice) and Casp3− groups (5 mice) 28 days after CFC. Unpaired *t*-test. (**i**) AAVs were injected to RSC as in Fig. 6j in ROSA-LSL-tdTomato mice. *Left*: Cell death in Casp3+ group resulted in lower tdT+ RSC cell density compared with Casp3− group. *Right*: tdT+ RSC cell density in Casp3+ (9 mice) and Casp3− groups (7 mice) 28 days after CFC. Unpaired *t*-test. Data are presented as the mean ± SEM in (c), (h), and (i) or as the mean ± 95% confidence interval in (g). Source data

These results raise the possibility that sustained activity of DG engram neurons may strengthen mPFC engram circuit and facilitate mPFC engram maturation during systems consolidation. To test this, we examined how the ablation of DG engram neurons affected the strengthening of mPFC engram circuit. In the Casp3^+^ group, DG engram neurons underwent Casp3-mediated cell death, and mice showed very weak freezing behavior during remote memory recall (Fig. 5g,h). Photostimulation of local ChR2^+^ mPFC engram inputs induced qEPSCs, which were recorded in tdT^+^ engram (E–E synapses) and tdT^−^ nonengram neurons (E–NE synapses) in the presence of Sr^2+^, TTX and 4-AP (Fig. 5i). In the Casp3^+^ group, the peak amplitude of evoked qEPSCs did not differ between engram versus nonengram neurons (Fig. 5i,j), indicating the absence of strengthening of local mPFC engram circuit. However, in the Casp3^−^ group, we observed the strengthening of local mPFC engram circuit (Fig. 5j). The ablation of DG engram neurons also inhibited the strengthening of interhemispheric mPFC engram circuit (Extended Data Fig. 8a–c). These results indicate the critical role of DG engram in the strengthening of mPFC engram circuits. Thus, sustained activity of DG engram after learning can contribute to remote memory consolidation possibly by strengthening mPFC engram circuits.

### RSC relays hippocampal signal to PFC engram

**Fig. 6:**
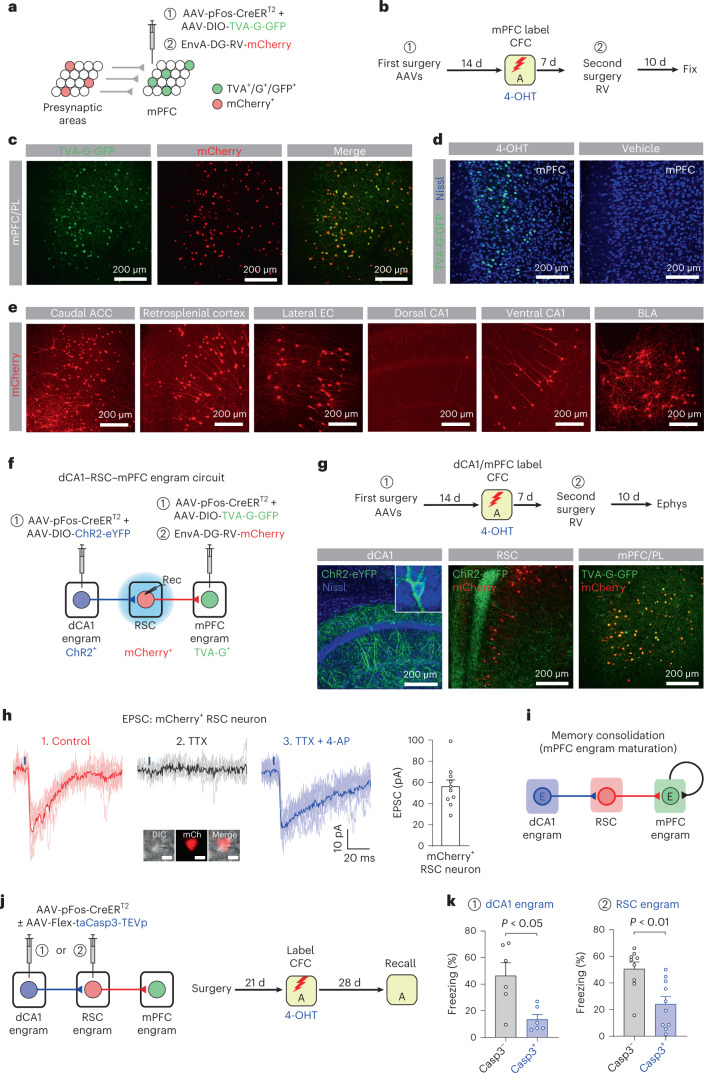
RSC connected hippocampal CA1 engram neurons to mPFC engram neurons. **a**, Experimental setup for **b**–**e**. mPFC engram neurons expressed TVA-G-GFP, whereas neurons monosynaptically projecting to mPFC engram neurons expressed mCherry. **b**, After the injection of AAV-pFos-CreER^T2^ and AAV-DIO-TVA-G-GFP into mPFC, mice underwent CFC and received 4-OHT injection. After 1 week, EnvA-ΔG-RV-mCherry was injected into mPFC. **c**, Images showing TVA-G-GFP + mPFC neurons (green) and RV-infected mCherry^+^ mPFC neurons (red). **d**, TVA-G-GFP (green) was expressed in mPFC engram neurons in mice that received 4-OHT but not vehicle injection after CFC. **e**, Images showing mCherry^+^ neurons in cACC, RSC, lateral EC, ventral CA1 and BLA. Note few mCherry^+^ neurons in dorsal CA1. **f**, Experimental setup for **g**–**h**. mPFC engram neurons expressed TVA-G-GFP, whereas dCA1 engram neurons expressed ChR2-eYFP. RSC neurons projecting to mPFC engram neurons expressed mCherry. **g**, Top: after injection of AAVs into mPFC and dCA1, mice underwent CFC and received RV injection into mPFC. Bottom: images showing ChR2-eYFP^+^dCA1 engram neurons (green, left) and TVA-G-GFP^+^ mPFC engram neurons (green, right). Middle panel shows mCherry^+^ RSC neurons (red) and ChR2-eYFP^+^ dCA1 axons (green). **h**, Left: traces of EPSCs induced by photostimulation of dCA1 engram inputs and recorded in mCherry^+^ RSC neurons at −80 mV (red). TTX completely blocked EPSCs (black). Subsequent 4-AP application in the presence of TTX rescued EPSCs (blue). Scale bar, 10 μm (inset). Right: plot of EPSC amplitudes in dCA1 engram inputs to mCherry^+^ RSC neurons. **i**, RSC connects dCA1 engram neurons to mPFC engram neurons. dCA1−RSC−mPFC engram circuit can contribute to remote memory consolidation. **j**, Experimental setup for **k**. dCA1 or RSC engram neurons active during CFC underwent Casp3-mediated cell death in Casp3^+^ but not Casp3^−^ group. Mice were tested for memory recall 28 d after CFC. **k**, Comparison of freezing behavior during remote memory recall between Casp3^+^ and Casp3^−^ groups for the ablation of dCA1 (left, six mice per group) and RSC engram (right, 10 mice for Casp3^+^ and 9 mice for Casp3^−^). Unpaired *t*-test. Data are presented as the mean ± s.e.m. Details of the statistical analyses are presented in Supplementary Table 1. Source data

Dorsal DG engram can contribute to systems consolidation by modulating mPFC activity possibly through the dorsal CA1 hippocampus (dCA1), the major output of the hippocampus. Consistent with this, the ablation of dCA1 engram neurons active during CFC prevented the strengthening of local mPFC engram circuit (Extended Data Fig. 8d–g). As the dCA1 weakly projects to the mPFC^28^, dCA1 signals may be conveyed to mPFC engram through intermediary areas during systems consolidation. To identify these areas, we examined presynaptic inputs to mPFC engram neurons, using rabies virus (RV)-mediated retrograde (rg) trans-synaptic tracing^29^. mPFC engram neurons expressed TVA-G-GFP and were infected with EnvA-expressing and G-deficient RV-mCherry, resulting in mCherry expression in neurons monosynaptically projecting to mPFC engram neurons (Fig. 6a–d). mCherry^+^ neurons were detected in the cACC, RSC, lateral entorhinal cortex (EC), ventral CA1 hippocampus and BLA, while few mCherry^+^ neurons were found in the dCA1 (Fig. 6e). As the RSC has been implicated in systems consolidation^20^, we examined whether the RSC might relay signals of dCA1 engram neurons to mPFC engram neurons. dCA1 engram neurons expressed ChR2-eYFP, while RSC neurons projecting to mPFC engram neurons were labeled with mCherry (Fig. 6f,g). Within the RSC, photostimulation of ChR2-eYFP^+^ dCA1 engram inputs induced monosynaptic EPSCs in mCherry^+^ RSC neurons (Fig. 6h), indicating that RSC neurons projecting to mPFC engram neurons received monosynaptic dCA1 engram inputs. These results suggest that a subset of RSC neurons could convey the signal of dCA1 engram to mPFC engram during remote memory consolidation (Fig. 6i). Consistent with this, the ablation of engram neurons in the dCA1 or RSC inhibited remote memory recall (Fig. 6j,k and Extended Data Fig. 8h,i), suggesting that the role of the dCA1−RSC−mPFC circuit in systems consolidation.

### mPFC–BLA engram circuits for remote fear memory recall

**Extended Data Fig. 9:**
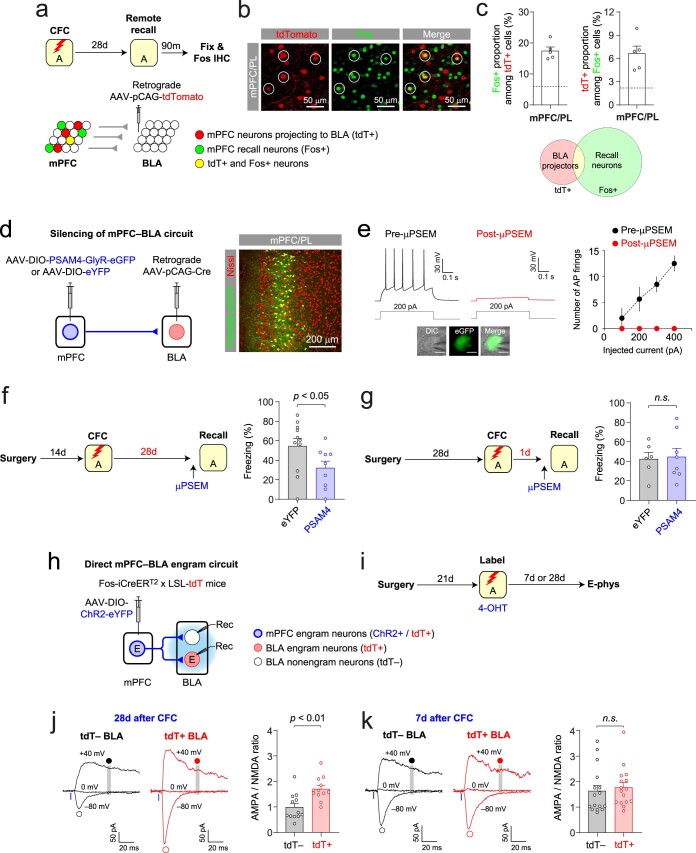
BLA−mPFC circuit was involved in remote fear memory recall, and mPFC−BLA engram circuit was strengthened during systems consolidation. (**a**) Experimental setup for (b, c). mPFC neurons projecting to BLA (BLA projectors) expressed tdTomato (tdT), while mPFC recall neurons were immunostained for c-Fos. (**b**) Images show tdT+ or Fos+ mPFC/PL neurons. Both tdT+ and Fos+ neurons are circled. (**c**) *Left*: proportion of mPFC recall neurons among BLA projectors (5 mice). *Right*: proportion of BLA projectors among mPFC recall neurons (5 mice). Dotted lines indicate the chance that randomly selected cells were Fos+ (left) or tdT+ (right). (**d**) *Left*: experimental setup for (e–g). BLA projectors in bilateral mPFC expressed PSAM4-GlyR-eGFP or eYFP. *Right*: image showing PSAM4-GlyR-eGFP-labeled mPFC neurons (green). (**e**) *Left*: AP firings before and after μPSEM application (10 μM) in the same PSAM4-GlyR-eGFP-expressing mPFC neuron (scale bar, 10 μm). *Right*: summary plot of AP firing (3 neurons). (**f**) *Left*: 28 days after CFC, mice were tested for memory recall after μPSEM injection. *Right*: freezing behavior during remote memory recall in PSAM4 (9 mice) and eYFP groups (11 mice). Unpaired *t*-test. (**g**) *Left*: a day after CFC, mice were tested for memory recall after μPSEM injection. *Right*: freezing behavior in PSAM4 (8 mice) and eYFP groups (6 mice). Unpaired *t*-test. (**h**) Experimental setup for (i–k). Photostimulation activated ChR2+ mPFC engram inputs and induced EPSCs in tdT− nonengram and tdT+ engram BLA neurons. (**i**) Electrophysiological experiments were 7 or 28 days after CFC without memory recall test. (**j**, **k**) *Left*: EPSCs in mPFC engram inputs to tdT− and tdT+ BLA neurons were recorded 28 days (j) or 7 days after CFC (k). *Right*: AMPA/NMDA EPSC ratio in tdT− and tdT+ BLA neurons (12 pairs in (j) and 16 pairs in (k), paired *t*-test). Data are presented as the mean ± SEM. Source data

A previous study suggests the critical role of the mPFC–BLA pathway in the recall of remote contextual fear memories^12^. Consistent with this, we found that 17.5 ± 1.3% of mPFC neurons projecting to the BLA were activated during remote fear memory recall, while 6.6 ± 1.0% of mPFC neurons active during memory recall projected to the BLA (mean ± s.e.m., five mice; Extended Data Fig. 9a–c). Moreover, silencing of mPFC neurons projecting to the BLA prevented the recall of remote but not recent contextual fear memory^30^ (Extended Data Fig. 9d–g), suggesting that activity in the mPFC–BLA pathway is required for remote fear memory recall.

**Fig. 7:**
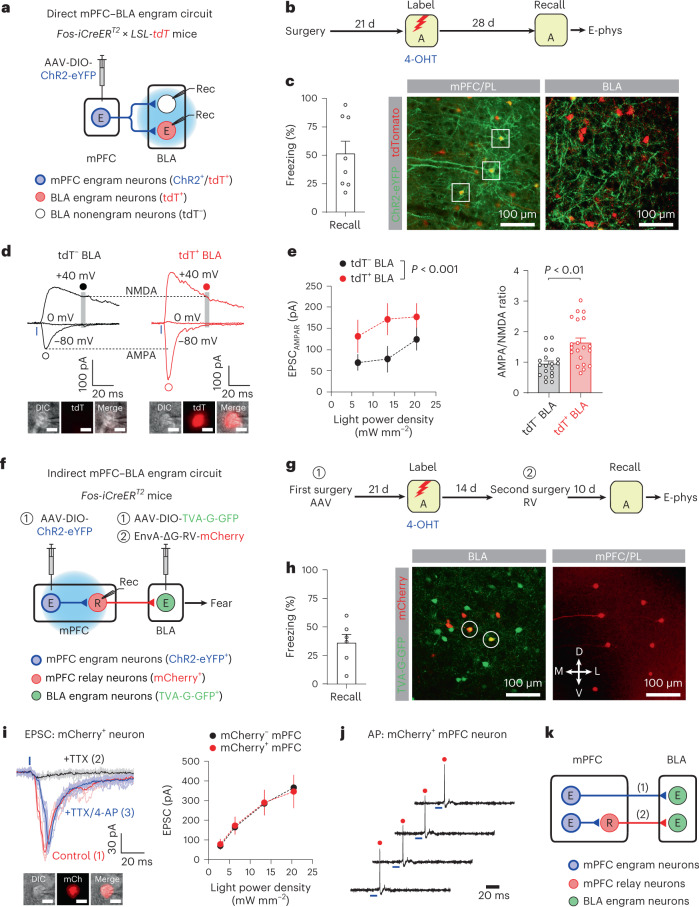
mPFC engram neurons were connected to BLA engram neurons through direct and indirect pathways for remote fear memory recall. **a**, Experimental setup for **b**–**e**. Photostimulation activated mPFC engram inputs and induced EPSCs in tdT^−^ nonengram and tdT^+^ engram BLA neurons. **b**, Mice were tested for memory recall 28 d after CFC. **c**, Left: freezing behavior during memory recall (eight mice). Middle: image showing ChR2-eYFP^+^/tdT^+^ mPFC neurons (squares). Right: image showing ChR2-eYFP^+^ mPFC axons (green) and tdT^+^ BLA neurons (red). **d**, Traces of EPSCs induced by photostimulation of mPFC engram inputs and recorded in tdT^−^ and tdT^+^ BLA neurons. AMPAR and NMDAR EPSCs were recorded as in Fig. 2d. Scale bar, 10 μm. **e**, Left: comparison of EPSC_AMPAR_ in 20 pairs of tdT^–^ versus tdT^+^ BLA neurons. Repeated measures two-way ANOVA. Right: comparison of AMPA/NMDA ratios of EPSCs in tdT^−^ (19 cells) versus tdT^+^ BLA neurons (20 cells). Unpaired *t*-test. **f**, Experimental setup for **g**–**j**. mPFC engram neurons expressed ChR2-eYFP, whereas BLA engram neurons expressed TVA-G-GFP. mPFC relay neurons (R, red) were labeled with mCherry. Photostimulation activated ChR2^+^ mPFC engram inputs and induced EPSCs in mPFC relay neurons. **g**, After AAV injection, mice underwent CFC and received RV injection. **h**, Left: freezing behavior during memory recall (six mice). Middle: BLA engram neurons were labeled with TVA-G-GFP and/or mCherry. TVA-G-GFP^+^/mCherry^+^ neurons are circled. Right: image showing mCherry^+^ mPFC relay neurons. **i**, Left: EPSCs were induced by photostimulation of mPFC engram inputs and recorded in mCherry^+^ mPFC relay neurons (red). TTX blocked EPSCs (black), which were rescued by 4-AP (blue). Scale bar, 10 μm. Right: comparison of EPSC amplitude in mCherry^+^ versus mCherry^−^ mPFC neurons (11 pairs) in the presence of TTX, 4-AP and SR-95531. **j**, Photostimulation (blue) of mPFC engram inputs induced AP firings (red) in mCherry^+^ mPFC relay neurons in cell-attached mode in the presence of SR-95531. **k**, mPFC engram neurons project to BLA engram neurons monosynaptically (1) or are connected to BLA engram neurons through mPFC relay neurons (2). Data are presented as the mean ± s.e.m. Details of the statistical analyses are presented in Supplementary Tables 1 and 2. Source data

We next examined how mPFC engram could activate BLA engram to induce remote fear memory recall^31^. mPFC engram neurons expressed ChR2-eYFP, and BLA engram neurons expressed tdT (Fig. 7a–c). Four weeks after CFC, photostimulation of ChR2^+^ mPFC engram inputs induced EPSCs in tdT^+^ BLA neurons (Fig. 7d), indicating that mPFC engram neurons monosynaptically projected to BLA engram neurons. Moreover, both AMPAR EPSC amplitude and the AMPA/NMDA ratio were substantially larger in tdT^+^ BLA neurons than in tdT^–^ neurons (Fig. 7d,e), suggesting stronger connections of mPFC engram inputs to BLA engram neurons than those inputs to nonengram neurons. The strengthening of the mPFC–BLA engram circuit was also observed without memory recall before recording, indicating that it was not induced by memory recall (Extended Data Fig. 9h–j). Moreover, synapse-specific strengthening of the mPFC–BLA engram circuit was not detected 7 d after CFC, suggesting that the engram circuit was progressively strengthened during systems consolidation (Extended Data Fig. 9k). With these synaptic changes, mPFC engram neurons can efficiently reactivate BLA engram neurons during remote fear memory recall.

As only a small fraction of mPFC engram neurons projected to the BLA (Extended Data Fig. 1g,h), we examined whether mPFC engram might indirectly activate BLA engram by recruiting mPFC neurons projecting to BLA engram neurons, which we termed ‘mPFC relay neurons’. We labeled mPFC relay neurons with mCherry using RV-mediated trans-synaptic tracing, while mPFC engram neurons expressed ChR2-eYFP (Fig. 7f–h). Photostimulation of ChR2^+^ mPFC engram neurons induced monosynaptic EPSCs and action potential firings in 88% and 40% of mPFC relay neurons examined, respectively (33 and 20 cells examined, respectively, Fig. 7i–j). Although mPFC relay neurons did not receive stronger mPFC engram inputs than other mPFC neurons (Fig. 7i), these results indicate that mPFC engram neurons can activate BLA engram neurons through mPFC relay neurons and contribute to remote fear memory recall (Fig. 7k).

### Strong connections between mPFC engram and recall neurons

**Fig. 8:**
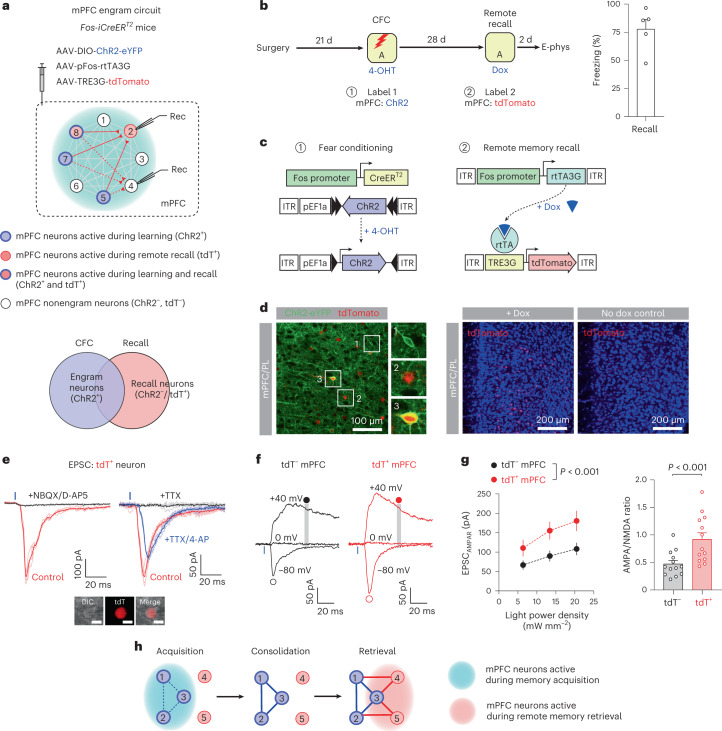
mPFC neurons active during initial learning were strongly connected to mPFC neurons recruited during remote memory recall. **a**, Experimental setup. mPFC engram neurons active during CFC expressed ChR2-eYFP (blue). mPFC recall neurons active during remote memory recall expressed tdT (red). **b**, Left: mice received 4-OHT injection after CFC (label 1). After 28 d, they received Dox injection and were tested for memory recall (label 2). Right: freezing behavior during remote memory recall (five mice). **c**, Left: mPFC neurons active during CFC expressed iCreER^T2^, resulting in 4-OHT-dependent recombination and ChR2 expression. Right: mPFC neurons active during remote memory recall expressed rtTA3G, resulting in tdT expression in the presence of Dox. **d**, Left: images showing mPFC neurons expressing ChR2-eYFP (green, 1), tdT (red, 2) or both (3). Right: mPFC recall neurons expressed tdT in Dox-injected mice. **e**, Traces of EPSCs induced by photostimulation of ChR2^+^ axons and recorded in tdT^+^/ChR2^−^ mPFC neurons (red) at −80 mV. Left: EPSCs were inhibited by NBQX and D-AP5 (black). Right: TTX blocked EPSCs (black), which were rescued by 4-AP (blue). Scale bar, 10 μm. **f**, Traces of EPSCs induced by photostimulation and recorded in tdT^–^/ChR2^–^ mPFC neuron (black) and tdT^+^/ChR2^−^ mPFC recall neurons (red). AMPAR EPSCs and NMDAR EPSCs were recorded as in Fig. 2d. TTX and 4-AP were added to isolate monosynaptic EPSCs. **g**, Left: comparison of the amplitude of EPSC_AMPAR_ recorded in tdT^–^/ChR2^–^ versus tdT^+^/ChR2^–^ neurons (15 pairs). Repeated measures two-way ANOVA. Right: comparison of AMPA/NMDA ratio between tdT^–^/ChR2^–^ versus tdT^+^/ChR2^–^ neurons (13 pairs). Paired *t*-test. **h**, Left: some mPFC neurons (neurons 1–3) are recruited during learning (memory acquisition). These engram neurons are weakly connected to one another. Middle: excitatory connections between mPFC engram neurons are strengthened during systems consolidation (blue lines). Right: remote memory recall reactivates some mPFC engram neurons (neuron 3), while it also activates mPFC recall neurons (neurons 4 and 5), which receive stronger inputs of mPFC engram neurons (red lines) than other mPFC neurons. Data are presented as the mean ± s.e.m. Details of the statistical analyses are presented in Supplementary Tables 1 and 2. Source data

**Extended Data Fig. 10:**
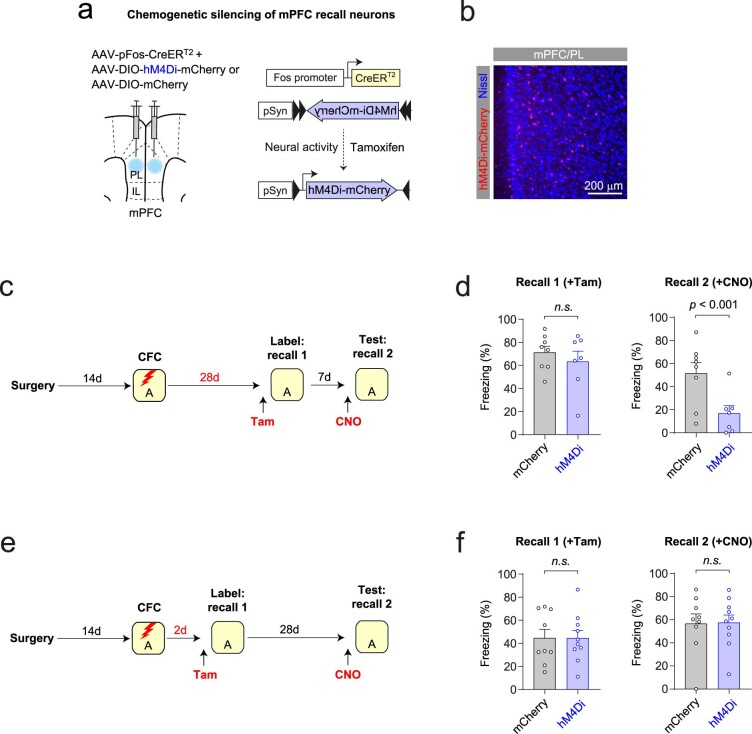
Remote memory recall was inhibited by chemogenetic silencing of mPFC neurons active during remote but not recent memory recall. (**a**) Experimental setup. A mixture of AAV-pFos-CreER^T2^ and AAV-DIO- hM_4_D_i_-mCherry (hM_4_D_i_ group) or AAV-DIO-mCherry (mCherry group) was bilaterally injected to the mPFC. mPFC neurons active during memory recall expressed hM_4_D_i_-mCherry or mCherry. (**b**) Image showing mPFC neurons expressing hM_4_D_i_-mCherry (red). Blue fluorescence indicates Nissl stain. (**c**) Four weeks after CFC, the mice receive a tamoxifen injection (Tam) to label mPFC neurons active during remote memory recall (recall 1) with hM_4_D_i_-mCherry or mCherry. A week after the recall 1 session, the mice received a CNO injection and were tested for memory recall in the same context (recall 2). (**d**) Summary plots showing the average freezing time during recall 1 and recall 2 sessions in the hM_4_D_i_ (7 mice) and mCherry groups (8 mice). Repeated measures two-way ANOVA with *post hoc* comparisons. (**e**) Two days after CFC, the mice receive a tamoxifen injection to label mPFC neurons active during recent memory recall (recall 1) with hM_4_D_i_-mCherry or mCherry. Four weeks after recall 1 session, the mice received a CNO injection and were tested for remote memory recall in the same context (recall 2). (**f**) Summary plots showing the average freezing time during recall 1 and recall 2 sessions in the hM_4_D_i_ (10 mice) and mCherry groups (9 mice). Repeated measures two-way ANOVA with *post hoc* comparisons. Data are presented as the mean ± SEM. Source data

While a substantial proportion (~30%) of mPFC engram neurons were reactivated during remote memory recall, other mPFC neurons were also recruited during memory recall (Fig. 1d). During remote memory recall, reactivated mPFC engram neurons may recruit other mPFC neurons that were not active during CFC, which termed ‘mPFC recall neurons’. To test this, we examined whether mPFC engram neurons were monosynaptically connected to mPFC recall neurons. Using dual independent labeling^2^, we independently labeled mPFC neurons active during CFC and those recruited during remote memory recall. After CFC and 4-OHT injection, mPFC engram neurons active during CFC expressed ChR2-eYFP (Fig. 8a–d). After 4 weeks, the mice received doxycycline (Dox) injection and were tested for remote memory recall, resulting in tdT expression in mPFC neurons active during recall (Fig. 8b–d). Thus, ChR2^+^ neurons represent mPFC engram neurons, whereas tdT^+^/ChR2^–^ neurons were mPFC recall neurons. Photostimulation of ChR2^+^ engram inputs induced glutamatergic and monosynaptic EPSCs in tdT^+^/ChR2^–^ mPFC neurons (Fig. 8e), indicating that mPFC recall neurons received excitatory inputs of mPFC engram neurons. Moreover, both AMPAR EPSCs and the AMPA/NMDA ratio were substantially larger in tdT^+^/ChR2^–^ mPFC neurons than in tdT^–^/ChR2^–^ neurons (Fig. 8f,g), indicating that mPFC recall neurons received stronger mPFC engram inputs than other neurons did. Thus, during remote memory recall, reactivated mPFC engram neurons can efficiently recruit mPFC recall neurons (Fig. 8h). Consistent with this, silencing of mPFC neurons active during remote but not recent memory recall inhibited subsequent remote memory recall (Extended Data Fig. 10).

## Discussion

Once acquired, contextual memories gradually mature to a stabilized form in the neocortex^3^. After systems consolidation, the retrieval of remote contextual memories requires neocortical activity and depends less on hippocampal activity^8,11,32–34^ (but see also refs. ^35,36^) as the standard consolidation model proposes. During systems consolidation, mPFC engram neurons slowly undergo enduring neuronal and synaptic changes for long-term memory storage. Although a previous study suggests that dendritic spine density is globally increased in mPFC engram neurons after systems consolidation^12^, synapse-specific substrates of remote contextual memories have not been identified. In this study, we demonstrate that the long-term storage of remote contextual memories involves progressive and synapse-specific strengthening of excitatory connections between mPFC engram neurons.

Previous studies suggest that learning rapidly generates neocortical memory engram^37,38^. Using activity-dependent labeling^39^, we tagged mPFC neurons recruited during CFC, which were more readily reactivated during remote memory recall than other mPFC neurons. Consistent with previous reports^10,12^, optogenetic activation of mPFC neurons active during CFC induced memory recall, suggesting that mPFC engram is generated early during learning. However, another study suggests that mPFC engram is more dynamic and continues to evolve after learning^40^.

Previous studies implicated neocortical synaptic plasticity in remote memory consolidation^41–43^. A subset of new dendritic spines induced by learning is preserved in neocortical neurons throughout life, supporting enduring memories^44^. Consistent with this, our study suggests that remote memory consolidation correlates with the strengthening of mPFC engram circuits. When examined 28 d after CFC, both interhemispheric and local excitatory connections between mPFC engram neurons displayed higher synaptic efficacy than those between engram and nonengram neurons or those between nonengram neurons, suggesting synapse-specific strengthening of mPFC engram circuits during systems consolidation. The strengthening of the engram circuit was also observed in the ACC but not in the RSC, although both areas are involved in remote memory consolidation^9,20^. The extent of BLA inputs conveying US signals during CFC may determine which neocortical area undergoes enduring synaptic changes during systems consolidation.

The strengthening of mPFC engram circuits was not detected 7 d after CFC, indicating that the engram circuits were progressively strengthened during systems consolidation. Moreover, silencing of mPFC engram neurons inhibited the retrieval of remote but not recent contextual memory, consistent with previous reports^10,12^. Progressive strengthening of mPFC engram circuits may account for the time-dependent role of mPFC engram in contextual memory recall. Moreover, the inhibition of CREB in mPFC engram neurons inhibited remote memory recall^10^. As the same manipulation also prevented the strengthening of mPFC engram circuit, neuronal and/or synaptic plasticity in mPFC engram neurons is likely involved in remote memory consolidation. The extinction of remote contextual memories weakened mPFC engram circuit, further supporting the correlation between remote memory recall performance and synaptic strength of mPFC engram circuit. As mPFC engram neurons are crucial for remote memory recall, the weakening of mPFC engram circuit after extinction can inhibit the retrieval of remote contextual memory. Our finding that the original engram is modified by extinction learning is consistent with previous reports^45,46^.

Our study highlights the role of sustained hippocampal activity in mPFC engram maturation. As mPFC activity is modulated during hippocampal sharp-wave ripples^47^, hippocampal signals can be conveyed to the mPFC, facilitating hippocampal–neocortical interactions during sleep for memory consolidation^48^. Consistent with this, both the hippocampus and mPFC show the replay of activity patterns representing memories during sleep^49,50^. In our study, ablation of DG engram neurons inhibited both remote memory recall and the reactivation of mPFC and BLA engram during recall. These findings suggest that sustained activity of DG engram may contribute to remote memory consolidation by facilitating mPFC engram maturation. Notably, the ablation of DG engram neurons also prevented the strengthening of mPFC engram circuits. Our study also demonstrates that RSC neurons projecting to mPFC engram neurons receive monosynaptic inputs from dCA1 engram neurons, forming the dorsal hippocampal–RSC–mPFC engram circuit. Moreover, the ablation of dCA1 or RSC engram neurons inhibited remote memory recall. Thus, the RSC can convey hippocampal signals to mPFC engram for systems consolidation. Consistent with this, a previous study suggests that the stimulation of RSC engram neurons facilitated systems consolidation by modulating mPFC activity^20^, highlighting the role of the RSC in remote memory consolidation.

Then, how does the strengthening of mPFC engram circuits contribute to the consolidation and retrieval of remote contextual memories? Although the medial entorhinal cortex (MEC) is necessary for mPFC engram generation, activity in the MEC–mPFC pathway is not required for remote memory recall^12^. As mPFC engram generated by the MEC is reactivated by distinct inputs during remote memory recall, these inputs likely activate only a fraction of mPFC engram neurons. In our study, excitatory connections between mPFC engram neurons were strengthened during remote memory consolidation, while mPFC engram neurons received weaker inhibitory engram inputs than other mPFC neurons. These synaptic mechanisms can facilitate the reactivation of mPFC engram during remote memory recall, even if only a fraction of mPFC engram neurons are directly reactivated by extrinsic inputs. This is analogous to the role of the strengthening of recurrent hippocampal CA3–CA3 synapses in pattern completion^51,52^. Moreover, the strengthening of mPFC engram circuits can also promote synchronized activity of mPFC engram neurons for remote memory recall^53,54^.

In our study, some mPFC neurons projecting to the BLA were reactivated by remote memory recall, and their silencing inhibited remote but not recent memory recall, indicating that the mPFC–BLA pathway is necessary for the recall of remote contextual fear memories^12^. Our results also suggest that mPFC engram neurons are connected to BLA engram neurons through monosynaptic and disynaptic pathways. Moreover, mPFC engram neurons were more strongly connected to BLA engram neurons than to BLA nonengram neurons when examined 28 d but not 7 d after CFC. With this strong connectivity, mPFC engram can efficiently activate BLA engram to generate defensive behaviors in a threat-predictive context during remote fear memory recall. Synapse-specific strengthening of the mPFC–BLA engram circuit may support the development of a schematic memory with few contextual details in extrahippocampal areas as the transformation theory proposes^13,14^.

While a subset of mPFC engram neurons was reactivated during remote memory recall, other mPFC neurons that were not active during initial learning were also recruited during remote memory recall. These mPFC recall neurons received stronger excitatory inputs of mPFC engram neurons than other mPFC neurons. Thus, mPFC engram neurons reactivated during memory recall can efficiently recruit mPFC recall neurons, contributing to remote memory recall. Consistent with this, silencing of mPFC neurons recruited during remote but not recent memory recall inhibited subsequent memory retrieval, highlighting the role of mPFC recall neurons in remote memory recall. Thus, the retrieval of remote contextual fear memory can be suppressed by inhibiting a subset of mPFC neurons tagged even after the contextual memory is consolidated in neocortical circuits, suggesting clinical implications in attenuating chronic maladaptive fear memory in posttraumatic stress disorder. Together, our study elucidates fundamental mechanisms by which remote contextual memories are consolidated in the neocortex.

## Methods

### Subjects

We obtained heterozygous *Fos-iCreER*^*T2*^ (TRAP2) mice by crossing wild-type C57BL6/J (Jackson Laboratory Stock, 000664) and *Fos-iCreER*^*T2*^^(+/+)^ mice (Jackson Laboratory Stock, 030323). We obtained *Fos-iCreER*^*T2*^^(+/−)^ × *ROSA-LSL-tdTomato*^(+/−)^ mice by crossing *Fos-iCreER*^*T2*^^(+/+)^ and Ai14 *ROSA-LSL-tdTomato*^(+/+)^ mice (Jackson Laboratory Stock, 007914). The mice were housed in home cages on a 12-h light/dark cycle at 23–25 °C with food and water continuously available. Humidity range was 30–70%. The light cycle was from 8 AM to 8 PM. Eight- to 12-week-old mice of both sexes were used for experiments. All of the animal procedures were approved by the Institutional Animal Care and Use Committee of the University of California, Riverside.

### Virus constructs

The recombinant adeno-associated viruses (AAVs) were packaged by Addgene, Vectorbuilder, the Vector Core at the University of North Carolina (UNC). The AAV titers were 1.7–3.0 × 10^13^ genome copies (GC) ml^–1^ for AAV5-pEF1α-DIO-hChR2(H134R)-eYFP (Addgene, 20298-AAV5), 0.9 × 10^12^ GC ml^–1^ for AAV5-pEF1α-DIO-eYFP (UNC), 0.9 × 10^12^ GC ml^–1^ for AAV5-pEF1α-DIO-mGFP-MutCREB(S133A) (Vectorbuilder; Addgene plasmid,194642), 2.8–5.5 × 10^11^ GC ml^–1^ for AAV5-pFos-CreER^T2^ (Vectorbuilder; Addgene plasmid,194643), 4.6 × 10^12^ GC ml^–1^ for AAV5-pEF1α-Flex-taCasp3-TEVp (UNC), 3.9–5.9 × 10^12^ GC ml^–1^ for AAV1-pSyn-DIO-TVA-G-GFP (UNC), 5.9 × 10^12^ GC ml^–1^ for AAV5-pFos-rtTA3G (Vectorbuilder; Addgene plasmid,120309), and 5.0 × 10^12^ GC ml^–1^ for AAV5-TRE3G-tdTomato (Vectorbuilder; Addgene plasmid,194644), 4.3 × 10^12^ GC ml^–1^ for AAV5-pCaMKII-eYFP (UNC), 3.8 × 10^12^ GC ml^–1^ for rg AAV2-pCAG-tdTomato (UNC), 5.0 × 10^10^ GC ml^–1^ for AAV5-pCaMKII-Cre-GFP (UNC), 2.4–3.1 × 10^13^ GC ml^–1^ for AAV5-pSyn-DIO-hM_4_D_i_-mCherry (Addgene,44362-AAV5), 1.0 × 10^12^ GC ml^–1^ for AAV5-pEF1α-DIO-mCherry (UNC), 2.5 × 10^13^ for AAV5-pSyn-DIO-PSAM4-GlyR-eGFP (Addgene,119741-AAV5), and 4.1 × 10^12^ GC ml^–1^ for rg AAV2-pCAG-Cre (UNC). RV (EnvA-ΔG-RV-mCherry) was obtained from the gene transfer, targeting and therapeutics core of the Salk Institute for Biological Studies, and the titer was 1.5–2.7 × 10^8^ transducing units per milliliter.

### Surgery

Before surgery, general anesthesia was induced by placing the mice in a transparent anesthetic chamber filled with 5% isoflurane. The anesthesia was maintained during surgery with 1.5% isoflurane applied to the nostrils of the mice using a precision vaporizer. Mice were checked for the absence of the tail-pinch reflex as a sign of sufficient anesthesia. The mice were then immobilized in a stereotaxic frame with nonrupture ear bars (David Kopf Instruments), and ophthalmic ointment was applied to prevent eye drying. After an incision was made along the midline of the scalp, unilateral or bilateral craniotomies were performed using a microdrill with 0.5 mm burrs. The tips of glass capillaries loaded with virus-containing solution were placed into the prelimbic division of the mPFC (mPFC/PL) (1.9 mm rostral to bregma, 0.4 mm lateral to the midline and 1.2 mm ventral to the pial surface), cACC (0.5 mm rostral to bregma, 0.4 mm lateral to the midline and 0.9 mm ventral to the pial surface), RSC (2.2 mm caudal to bregma, 0.5 mm lateral to the midline and 0.6 mm ventral to the pial surface), BLA (1.5 mm caudal to bregma, 3.3 mm lateral to the midline and 3.4 ventral to the pial surface), DG (2.0 mm caudal to bregma, 1.1 mm lateral to the midline and 2.0 mm ventral to the pial surface) or dCA1 (2.0 mm caudal to bregma, 1.5 mm lateral to the midline and 1.2 mm ventral to the pial surface). Virus-containing solution was injected at a rate of 0.1 μl min^−1^ using a 10 μl Hamilton microsyringe and a syringe pump.

The total volume of injected virus-containing solution was 1.0 μl for AAV5-pEF1α-DIO-hChR2(H134R)-eYFP, 0.3–1.0 μl for AAV5-pEF1α-DIO-eYFP, 1.0 μl for AAV-pEF1α-DIO-mGFP-MutCREB(S133A), 0.5–1.0 μl for AAV1-pSyn-DIO-TVA-G-GFP, 0.5 μl for AAV5-pCaMKII-GFP and 1.0 μl for rg AAV2-pCAG-tdTomato. In Fig. 5 and Extended Data Fig. 8a–c, a mixture of AAV5-pFos-CreER^T2^ (0.25 μl) and AAV5-pEF1α-Flex-taCasp3-TEVp (0.25 μl) was bilaterally injected into the dorsal DG. In Fig. 6a–h, a mixture of AAV5-pFos-CreER^T2^ (0.5 μl) and AAV1-pSyn-DIO-TVA-G-GFP (0.5 μl) into the mPFC/PL during the first virus injection surgery. In Fig. 6f–h, a mixture of AAV5-pFos-CreER^T2^ (0.5 μl) and AAV5-pEF1α-DIO-hChR2(H134R)-eYFP (0.5 μl) was also injected into the dorsal CA1 during the first virus injection surgery. In Fig. 6j,k and Extended Data Fig. 8h,i, a mixture of AAV5-pFos-CreER^T2^ (0.25 μl) and AAV5-pEF1α-Flex-taCasp3-TEVp (0.25 μl) was bilaterally injected into the dCA1 or RSC. In Fig. 8, a mixture of AAV5-pEF1α-DIO-hChR2(H134R)-eYFP (0.33 μl), AAV5-pFos-rtTA3G (0.33 μl) and AAV5-TRE3G-tdTomato (0.33 μl) was injected into the mPFC/PL. In Extended Data Fig. 4, a mixture of AAV5-pEF1α-DIO-hChR2(H134R)-eYFP (0.4 μl) and AAV5-pCaMKII-Cre-GFP (0.2 μl) was injected into the mPFC/PL. In Extended Data Fig. 8d–g, a mixture of AAV5-pFos-CreER^T2^ (0.25 μl) and AAV5-pEF1α-Flex-taCasp3-TEVp (0.25 μl) was bilaterally injected into the dCA1. After injection, the capillary was left in place for an additional 5 min to allow diffusion of the virus solution and then withdrawn. The scalp incision was closed with surgical sutures, and the mice were given a subcutaneous injection of buprenorphine-containing saline (1 ml, 0.12 mg buprenorphine per kilogram body weight) for postoperative analgesia and hydration.

For the experiments described in Fig. 1h–l, AAV-DIO-ChR2-eYFP (ChR2 group) or AAV-DIO-eYFP (eYFP group) was bilaterally injected into the mPFC/PL in *Fos-iCreER*^*T2*^ mice, and a dual fiberoptic cannula (200 μm in diameter, numerical aperture of 0.53; Doric Lenses, DFC_200/245-0.53_3mm_DF0.9_FLT) was implanted dorsal to the mPFC/PL (1.7 mm rostral to bregma and 0.8 mm ventral to the pial surface) and secured with dental cement. To minimize light leakage during photostimulation, which can act as a visual cue, we painted all optical pathways, including the dental cement securing the cannula, with black nail polish. We verified the cannula implantation site in each animal.

### Contextual fear conditioning

Mice were singly housed in their HCs a day before CFC. On the training day, mice were placed in Context A (dimension, 30 cm × 24 cm × 21 cm; stainless steel grid floor, white acrylic flat wall with black vertical stripes, white light illumination and benzaldehyde odor) within a fear conditioning chamber (Med Associates) between 9:30 AM and 10:30 AM. After 3 min, the mice received the first US (electric footshock, 0.5 mA, 2 s duration) and were given four more US with a 2-min interval except in Fig. 3e,f, in which the mice received total of two USs for CFC. The temperature in the fear conditioning chamber was 23–25 °C. One day after CFC, the mice were group-housed until 2 d before remote memory recall test. For the recall of contextual fear memories, the mice were exposed to Context A for 5 min between 9:30 AM and 10:30 AM after 1 min of acclimatization in Context A. Freezing behavior was quantified as the percentage of time immobile. Immobility for more than 2 s was counted as freezing behavior. The movement of the mice in the fear conditioning chamber was recorded using a near-infrared camera and analyzed with EthoVision XT 11 software (Noldus). In Extended Data Fig. 1a,b, some mice were trained and tested for freezing behavior in Context B (dimension, 30 cm × 24 cm × 21 cm; stainless steel grid floor, white acrylic curved wall with black horizontal stripes, white illumination and acetic acid odor).

### Extinction training

In Fig. 3g–j, the mice underwent memory extinction training 28 d after CFC and were exposed to Context A for 6 min once a day for 5 d (days 1–5). For each extinction training session, freezing behavior was quantified as the percentage of time immobile in Context A after 1 min of acclimatization. Electrophysiological recordings were performed within 1 h after the last (fifth) session of the extinction training on day 5.

### Activity-dependent neuronal labeling

To label neurons active during CFC, we fear conditioned *Fos-iCreER*^*T2*^ mice, *Fos-iCreER*^*T2*^ × *ROSA-LSL-tdTomato* mice or *ROSA-LSL-tdTomato* mice 2–3 weeks after virus injection surgery as described above. In some experiments (Figs. 5 and 6 and Extended Data Figs. 3, 8 and 10), we injected AAV-pFos-CreER^T2^ into the mPFC, DG, dCA1 or RSC to increase labeling efficiency. To open labeling window, the mice received an intraperitoneal injection of 4-OHT (H6278, Sigma-Aldrich; 50–75 mg kg^–1^ of body weight) under brief anesthesia with 5% isoflurane in the anesthesia chamber 10 min after CFC. 4-OHT was dissolved in DMSO (40 mg ml^–1^) and further dissolved in saline containing 2% TWEEN 80 in a water bath at 40–42 °C, resulting in 1 mg ml^–1^ 4-OHT solution. To minimize neuronal labeling by background noise, mice were kept in their HCs in a quiet place for 8 h before and after 4-OHT injection.

To independently labeled mPFC neurons active during CFC and those recruited during remote memory recall in Fig. 8, we injected a mixture of AAV-DIO-ChR2-eYFP (0.33 μl), AAV-pFos-rtTA3G (0.33 μl) and AAV-TRE3G-tdTomato (0.33 μl) into the mPFC/PL in *Fos-iCreER*^*T2*^ mice. After CFC, the mice received a 4-OHT injection (50 mg kg^–1^ of body weight) to label mPFC engram neurons with ChR2-eYFP. Four weeks after CFC, the mice received an intraperitoneal injection of doxycycline hyclate (Dox; D9891, Sigma-Aldrich; 50–100 mg kg^–1^ of body weight) dissolved in saline at 5 mg ml^–1^ under brief anesthesia with 5% isoflurane and returned to the HCs. One hour after Dox injection, the mice were exposed to Context A for the recall of remote fear memories for 5 min.

In Extended Data Fig. 10, mice received an intraperitoneal injection of tamoxifen (100 mg kg^–1^ of body weight, Sigma-Aldrich, T5648). Tamoxifen was dissolved in corn oil (Sigma-Aldrich, C8267) at 20 mg ml^–1^ with nutation for 6 h in the dark at room temperature (23–25 °C). Sixteen hours after tamoxifen injection, the mice were tested for the recall of recent or remote contextual fear memory in Context A for 2 min.

### In vivo optogenetic stimulation of mPFC engram neurons

In Fig. 1h–l, we injected AAV-pEF1α-DIO-ChR2-GFP (ChR2 group) or AAV-pEF1α-DIO-YFP (eYFP group) into the mPFC in *Fos-iCreER*^*T2*^ mice. A dual fiberoptic cannula was implanted dorsal to the mPFC/PL to illuminate mPFC neurons expressing ChR2-eYFP or eYFP. Three weeks after surgery, the mice were fear conditioned in Context A and received an intraperitoneal injection of 4-OHT 10 min later as described above. Four weeks after CFC, the optical cannula was connected to the optical cable (Doric Lenses, BFP(2)_200/220/900-0.53_0.42m_FCM-DF0.9) under brief anesthesia with 5% isoflurane. Immediately after cannula–cable connection, the mice were placed in Context B, which was the modified HC (dimension, 28 cm × 18 cm × 12 cm) placed within the fear conditioning chamber illuminated with red dim light. After full recovery from anesthesia, the activity of the mice was monitored for 3 min as the prestimulation baseline. Then, 5 Hz pulses of blue light illumination (3 ms pulses, 20 mW measured at each cannula tip) with a 450 nm laser (Opto Engine, MDL-III-450-200 mW) were applied to the mPFC/PL through an implanted optical cannula for 3 min (20-s laser on/10-s laser off, total 6 cycles) to reactivate mPFC engram neurons active during CFC. After 3 min photostimulation, the activity of the mice was monitored for 3 min as the poststimulation baseline. The mice underwent the behavioral test once per day for 3 d. In each mouse, freezing scores in the presence or absence of blue light illumination were calculated on each test day and averaged. Freezing score in the absence of blue light was calculated as the average freezing score of the pre- and poststimulation baselines.

### Chemogenetic silencing of mPFC neuronal populations

To silence the activity of mPFC engram neurons active during CFC in Extended Data Fig. 3, we bilaterally injected a 1:1 mixture of 0.5 μl AAV-pFos-CreER^T2^ (5.5 × 10^11^ GC ml^–1^) and 0.5 μl AAV-pSyn-DIO-hM_4_D_i_-mCherry (2.4–3.1 × 10^13^ GC ml^–1^, hM_4_D_i_ group) or AAV- pEF1α-DIO-mCherry (1.0 × 10^12^ GC ml^–1^, mCherry group) into the mPFC/PL. Two weeks after surgery, mice were placed in Context A within a fear conditioning chamber on the training day. After 3 min, the mice received the first US (electric footshock, 0.5 mA, 2 s duration) and were given one more US with a 2 min interval. After 10 min, the mice received an intraperitoneal injection of 4-OHT under brief anesthesia. In Extended Data Fig. 3d, we habituated the mice to handling and intraperitoneal injection by injecting saline (0.3 ml) once per day for 2 d (26 and 27 d after CFC). On the test day (28 d after CFC), the mice received an intraperitoneal injection of clozapine N-oxide hydrochloride (CNO; 3 mg kg^–1^ of body weight, dissolved in saline; Sigma-Aldrich, SML2304). After 45–60 min, the mice were placed in Context A and tested for the recall of remote contextual fear memory. After 1 min acclimatization, the activity of the mice was monitored for 5 min and freezing scores were calculated. In Extended Data Fig. 3e, the mice received a CNO injection 7 d after CFC and were tested for the recall of recent contextual fear memory.

To silence the activity of mPFC neurons active during remote memory recall in Extended Data Fig. 10a–d, we bilaterally injected a 1:1 mixture of 0.5 μl AAV-pFos-CreER^T2^ and 0.5 μl AAV-pSyn-DIO-hM_4_D_i_-mCherry (hM_4_D_i_ group) or AAV-pEF1α-DIO-mCherry (mCherry group) into the mPFC/PL. Two weeks after surgery, mice were placed in Context A within a fear conditioning chamber on the training day. After 3 min, the mice received the first US (electric footshock, 0.5 mA, 2 s duration) and were given two more US with a 2-min interval. Four weeks after CFC, the mice received an intraperitoneal injection of tamoxifen (100 mg kg^–1^ of body weight). After 16 h, the mice were tested for the recall of remote contextual fear memory in Context A for 2 min (recall 1). After 5 d, the mice received an intraperitoneal injection of saline (0.3 ml) once per day for 2 d for habituation. On the test day (7 d after recall 1), the mice then received an intraperitoneal injection of CNO hydrochloride (3 mg kg^–1^ of body weight). After 45–60 min, the mice were tested for the recall of remote contextual fear memory (recall 2). After 1 min acclimatization in Context A, the activity of the mice was monitored for 5 min and freezing scores were calculated. To silence the activity of mPFC neurons active during recent memory recall in Extended Data Fig. 10e,f, mice underwent the same surgery and were fear conditioned in Context A as described above. Thirty-two hours after CFC, the mice received an intraperitoneal injection of tamoxifen (100 mg kg^–1^ of body weight). After 16 h, the mice were tested for the recall of recent contextual fear memory in Context A for 2 min (recall 1). After 4 weeks, we habituated the mice to handling and intraperitoneal injection by injecting saline (0.3 ml) once per day for 2 d (28 and 29 d after CFC). On the test day (30 d after CFC), the mice received an intraperitoneal injection of CNO hydrochloride (3 mg kg^–1^ body weight). After 45–60 min, the mice were acclimatized in Context A for 1 min and tested for the recall of remote contextual fear memory (recall 2) for 5 min.

To silence the activity of mPFC neurons projecting to the BLA in Extended Data Fig. 9d–f, we bilaterally injected 0.5–1.0 μl AAV-pCAG-Cre (4.1 × 10^12^ GC ml^–1^) into the BLA and 1.0 μl AAV-pSyn-DIO-PSAM4-GlyR-eGFP (2.5 × 10^13^ GC ml^–1^, PSAM4 group) or AAV-pEF1a-DIO-eYFP (0.9 × 10^12^ GC ml^–1^, eYFP group) into the mPFC/PL. Two weeks after surgery, mice were placed in Context A on the training day. After 3 min, the mice received the first US (electric footshock, 0.5 mA, 2-s duration) and were given four more US with a 2-min interval. Four weeks after CFC, the mice received an intraperitoneal injection μPSEM 792 hydrochloride (μPSEM, 1 mg kg^–1^ of body weight, dissolved in saline; Tocris, 6865). After 60 min, the mice were tested for the recall of remote contextual fear memory for 5 min after 1 min acclimatization. In Extended Data Fig. 9g, mice were fear conditioned in Context A four weeks after virus injection surgery. One day after CFC, the mice received an intraperitoneal injection of μPSEM (1 mg kg^–1^ body weight) and were tested for the recall of remote contextual fear memory in Context A 60 min later.

### RV-mediated retrograde trans-synaptic tracing

In Fig. 6a–h, we first injected a 1:1 mixture of 0.5 μl AAV-pFos-CreER^T2^ (5.5 × 10^11^ GC ml^–1^) and 0.5 μl AAV-pSyn-DIO-TVA-G-GFP (3.9–5.9 × 10^12^ GC ml^–1^) into the mPFC/PL. After 2 weeks, the mice were fear conditioned in Context A and received an intraperitoneal injection of 4-OHT (50 mg kg^–1^ of body weight) 10 min later to label mPFC engram neurons with TVA-G-GFP. After 7 d, we injected 1.0 μl EnvA-ΔG-RV-mCherry (1.5–2.7 × 10^8^ transducing units per ml) into the mPFC/PL, which infected TVA/G-labeled mPFC neurons and propagated trans-synaptically, resulting in mCherry expression in neurons monosynaptically projecting to mPFC engram neurons. After 10 d, brain slices were prepared for electrophysiological or histological analysis.

In Fig. 7f–j, we first injected 1.0 μl AAV-pSyn-DIO-TVA-G-GFP into the BLA in *Fos-iCreER*^*T2*^ mice. After 3 weeks, the mice were fear conditioned in Context A and received a 4-OHT injection 10 min later to label BLA engram neurons with TVA-G-GFP. After 2 weeks, we injected 1.0 μl EnvA-ΔG-RV-mCherry into the BLA, which infected TVA/G-expressing BLA engram neurons and propagated trans-synaptically, resulting in mCherry expression in mPFC relay neurons. After 10 d, brain slices were prepared for electrophysiological recordings.

### Histology, microscopic imaging and cell counting

Acute brain slices (300 μm thick) were prepared with a vibratome (VT-1000S, Leica Biosystems) and fixed in 4% paraformaldehyde in PBS (137 mM NaCl, 2.7 mM KCl, 11.9 mM phosphate, pH 7.4) at room temperature for an hour. After fixation, slices were washed in PBS containing 0.3 % Triton X-100 (PBS-T) for 10 min and permeabilized in PBS-T at room temperature overnight. For Nissl staining, slices were incubated with Neurotrace fluorescent Nissl stain (1:40 diluted in PBS; N21479 and N21482, Thermo Fisher Scientific) for 3 h at room temperature and washed in PBS-T three times for 10 min each. After a final wash in PBS-T, Vectashield mounting medium (H-1200, Vector Laboratories) was applied to the slices, which were then covered with coverslips. Microscopic images were captured using the Leica TCS SP5 confocal system (Leica Microsystems). Images captured with different fluorescent channels were merged using ImageJ software (National Institute of Mental Health). For each mouse, the virus injection site was verified by the expression of fluorescent markers. Mice in which the target area was missed were excluded from the analysis. In Fig. 5c and Extended Data Fig. 8h,i, we captured confocal microscopic images of four representative fields (0.56 mm^2^ each) per mouse within the dorsal DG, dorsal CA1 or RSC, where tdT^+^ cells were distributed most densely. We manually counted tdT^+^ cells in confocal microscopic images and calculated the tdT^+^ cell density.

### c-Fos immunohistochemistry and analysis

Brain slices of the mPFC or BLA (150 μm thick) were prepared with the vibratome and fixed 90 min after remote memory recall test in Figs. 1d–g and 5e,f and Extended Data Fig. 9a–c. After fixation in 4% paraformaldehyde in PBS for an hour, the slices were permeabilized in PBS-T at room temperature for 2 d. Brain sections were then blocked with PBS containing 5% goat serum at 4 °C for an hour. The slices were washed with PBS-T for 10 min and incubated with a polyclonal affinity purified rabbit anti-c-Fos antibody (1:1,000 diluted in PBS-T; 226003, Synaptic Systems) at room temperature for 24 h. The slices were then washed with PBS-T three times for 10 min each and incubated with goat antirabbit IgG antibody-Alexa Fluor 647 (1:200 in PBS-T; A-21246, Thermo Fisher Scientific) at 4 °C for 24 h. The slices were then washed three times with PBS-T for 10 min each and mounted on glass slides for confocal microscopic imaging. For each mouse, we captured z-series confocal microscopic images of four representative fields (0.56 mm^2^ each) of the mPFC/PL or the BLA and z-stacked the images using ImageJ software. We manually counted tdT^+^ neurons and tdT^+^/c-Fos^+^ neurons in the mPFC and BLA. c-Fos^+^ cells were counted using Imaris 9 software (Bitplane). We then calculated the proportion of c-Fos^+^ neurons among all tdT^+^ neurons in each mPFC and BLA field and averaged the proportions for each mouse.

### Whole-cell patch-clamp recording in brain slices

For electrophysiological recording in brain slices, mice were deeply anesthetized with 5% isoflurane and decapitated. Brains were dissected quickly and chilled in ice-cold artificial cerebrospinal fluid (ACSF) containing 130 mM NaCl, 2.5 mM KCl, 2.5 mM CaCl_2_, 1 mM MgSO_4_, 1.25 mM NaH_2_PO_4_, 26 mM NaHCO_3_ and 10 mM glucose with 95% O_2_ and 5% CO_2_. Coronal brain slices (300 μm thick) were prepared with the vibratome. After a 1-h recovery at room temperature, slices were placed in the recording chamber and continuously perfused with ACSF at a rate of 1 ml per minute. The patch electrodes (1.5–2.2 MΩ resistance) were filled with pipette solution containing 140 mM Cs-methanesulfonate, 5 mM NaCl, 1 mM MgCl_2_, 10 mM HEPES, 0.2 EGTA, 2 mM MgATP, 0.5 mM NaGTP and 5 mM QX-314 chloride (Sigma-Aldrich, L1663) (290 mOsm, adjusted to pH 7.3 with CsOH). Whole-cell patch-clamp recordings were performed using a Multiclamp 700B amplifier, a Digidata 1550 digitizer, and Clampex 10 software (Molecular Devices). The temperature of recording chamber was carefully monitored to be 30–32 °C. The membrane potential was held constant at –80 mV in the voltage-clamp mode unless otherwise indicated. The liquid junction potential of 8.9 mV was corrected. Series (access) resistance was not compensated. Offline data analysis was performed using Clampfit 11 (Molecular Devices).

In Figs. 2, 4, 5g–j and 7 and Extended Data Figs. 4, 5 and 7, we prepared brain slices for electrophysiological recording within 1 h after memory recall test to minimize the effect of memory recall on synaptic strength of the engram circuits. In Fig. 8, we performed electrophysiological experiments 2 d after the remote memory recall session for sufficient tdT expression in mPFC recall neurons. In Extended Data Fig. 3c and 9e, the patch electrodes (2.5–3.0 MΩ resistance) were filled with solution containing 150 mM K-gluconate, 5 mM NaCl, 1 mM MgCl_2_, 10 mM HEPES, 0.2 EGTA, 2 mM MgATP and 0.5 mM NaGTP (290 mOsm, adjusted to pH 7.3 with KOH) and AP firings were induced with square pulses of depolarizing currents (100–400 pA with the increment of 100 pA, 0.5 s duration) in the current-clamp mode.

#### Photostimulation in brain slices

A blue collimated light-emitting diode (LED) with a peak wavelength of 470 nm (M470L3, Thorlabs) was used for photostimulation of ChR2-expressing axons. The LED was connected to the amplifier and digitizer through an LED driver (LEDD1B, Thorlabs). Brain slices in the recording chamber were illuminated through a ×40 water-immersion objective lens (Olympus LUMPLFLN 40XW) and a 450–490 nm filter (Chroma). The illumination area was 0.17 mm^2^ and was centered at the soma of the neuron patched for recording. The intensity and duration of photostimulation were controlled using a Digidata 1550 digitizer and pClamp 10 software (Molecular Devices). Light power in milliwatts (mW) was measured at 470 nm using a power meter (PM100A, Thorlabs) placed under the objective lens, and light power density (milliwatt/square millimeter (mW mm^–^^2^)) was calculated by dividing light power by illumination area. To evoke synaptic responses by photostimulation, we illuminated the slices every 20 s with blue light pulses of 1–5 ms duration (2.8–20.5 mW mm^–^^2^). When apparent polysynaptic activity was detected in EPSC recordings, we reduced the photostimulation intensity to prevent polysynaptic components from contributing to our measurement of AMPAR and NMDAR EPSC amplitudes. When we could not eliminate polysynaptic activity by adjusting the stimulation intensity, we terminated the experiments for the recorded neurons. Although the average peak amplitude of AMPAR EPSCs was relatively small in some neurons in Fig. 2f–h and Extended Data Fig. 2i–k, the EPSCs were reliably induced over repeated photostimulations in most mPFC neurons (Extended Data Fig. 2l). Only 26.3% of all mPFC neurons examined in these data sets (15 of total 57 neurons examined) displayed probabilistic EPSC with an average failure rate of 37.8 ± 4.8% (mean ± s.e.m.; Extended Data Fig. 2m). We excluded failures in our analysis of AMPAR EPSC and the AMPA/NMDA EPSC ratio.

#### AMPA/NMDA EPSC ratio

AMPAR EPSCs were recorded at −80 mV, and NMDAR EPSCs were recorded at +40 mV in voltage-clamp mode. SR-95531 (10 μM; Sigma-Aldrich, S106), a GABA-A receptor antagonist, was added to the ACSF to prevent contamination from IPSCs. For each neuron, the same photostimulation intensity and duration were used to record AMPAR and NMDAR EPSCs. To calculate the AMPA/NMDA EPSC ratio, we recorded the first set of AMPAR EPSCs (three to five traces) at −80 mV and then recorded NMDAR EPSCs (three to five traces) at +40 mV. Then, the holding potential was returned to −80 mV to record the second set of AMPAR EPSCs (three to five traces). We also recorded EPSCs at 0 mV. This recording protocol minimized the effect of time-dependent EPSC changes on the AMPA/NMDA ratio. To quantify AMPAR EPSCs, we averaged AMPAR EPSC traces recorded before and after the recording of NMDAR EPSCs and calculated the peak amplitude of averaged AMPAR EPSCs. To quantify NMDAR EPSCs, we averaged NMDAR EPSC traces and measured the mean amplitudes of the averaged NMDAR EPSCs between 47.5 ms and 52.5 ms after the onset of photostimulation. Then, we calculated the amplitude ratio of AMPAR EPSCs to NMDAR EPSCs. When the peak amplitude of AMPAR EPSCs was compared between groups, AMPAR EPSCs were induced by the photostimulation of the same light intensity (20.5 mW mm^–^^2^).

#### Evoked and spontaneous quantal EPSCs and IPSCs

To induce asynchronous release of glutamate from presynaptic terminals in Fig. 4d,e,h,i, Fig. 5i,j, Extended Data Fig. 6c,d, Extended Data Fig. 6g–i and Extended Data Fig. 8d–g, 2.5 mM CaCl_2_ in the ACSF was replaced with 4 mM SrCl_2_. Tetrodotoxin citrate (TTX, 1 μM; Tocris Bioscience, 1069) and 4-aminopyridine (4-AP, 1 mM; Tocris Bioscience, 0940) were added to the ACSF to prevent polysynaptic EPSCs. Photostimulation with blue light was applied to activate presynaptic axons of mPFC engram neurons and induce monosynaptic EPSCs, which were recorded at −80 mV in voltage-clamp mode in pairs of tdT^+^ and adjacent tdT^–^ neurons. EPSC traces recorded 0.5–1.5 s after photostimulation were analyzed to reliably measure individual qEPSCs optimally separated from one another. We used the event detection and template search function of Clampfit 11 software to detect photostimulation-evoked quantal EPSCs (evoked qEPSCs) in traces recorded 0.5–1.5 s after photostimulation. We manually verified each event detected by the software. In Fig. 4f,g and Extended Data Fig. 6e,f, spontaneous mEPSCs were recorded at −80 mV for 3 min without photostimulation in the presence of 1 μM TTX in pairs of tdT^+^ and adjacent tdT^–^ neurons.

To induce asynchronous release of GABA from presynaptic terminals in Extended Data Fig. 7, 2.5 mM CaCl_2_ in the ACSF was replaced with 4 mM SrCl_2_. TTX and 4-AP were added to the ACSF to prevent polysynaptic IPSCs. NBQX (10 μM; Tocris Bioscience, 1044) was also added to block excitatory glutamatergic transmission. Photostimulation with blue light was applied to activate presynaptic axons of local GABAergic mPFC engram neurons and induce monosynaptic quantal IPSCs (qIPSCs), which were recorded at 0 mV in voltage-clamp mode in pairs of tdT^+^ and adjacent tdT^–^ neurons. Photostimulation-evoked qIPSCs (evoked qIPSCs) were detected in traces recorded 0.5–1.5 s after photostimulation, using Clampfit 11 software. We manually verified each event detected by the software.

### Reproducibility

Micrographic images presented in the following figures are representative ones from experiments repeated independently: Fig. 1d (7 times), Fig. 1f (6 times), Fig.1j (13 times), Fig. 2b (22 times), Fig. 3b (10 times), Fig. 3f (15 times), Fig. 4c (14 times), Fig. 5c (11 times), Fig. 5e (9 times), Fig. 5f (9 times), Fig. 6c–e (5 times), Fig. 6g (5 times), Fig. 7c (8 times), Fig. 7h (6 times) and Fig. 8d (7 times) and Extended Data Fig. 1c (3 times), Extended Data Fig.1d (6 times), Extended Data Fig.1f (5 times), Extended Data Fig.1h (5 times), Extended Data Fig.1i (5 times), Extended Data Fig. 2b (12 times), Extended Data Fig. 3b (19 times), Extended Data Fig. 5c (4 times), Extended Data Fig. 5h (4 times), Extended Data Fig. 8h (5 times), Extended Data Fig. 8i (7 times), Extended Data Fig. 9b (5 times), Extended Data Fig. 9d (10 times) and Extended Data Fig.10b (12 times).

### Statistical analysis

Data are presented as the means ± s.e.m. unless indicated otherwise. For statistical comparisons, we used Welch’s *t*-test or ordinary or repeated measures ANOVA. For post hoc analysis, we used Bonferroni’s simultaneous multiple comparisons. All statistical tests were two-sided. Statistical analysis was performed with Minitab 21 software (Minitab), and *P* < 0.05 was considered statistically significant. Details of the statistical analyses are summarized in Supplementary Tables 1 and 2.

### Reporting summary

Further information on research design is available in the Nature Portfolio Reporting Summary linked to this article.

## Online content

Any methods, additional references, Nature Portfolio reporting summaries, source data, extended data, supplementary information, acknowledgements, peer review information; details of author contributions and competing interests; and statements of data and code availability are available at 10.1038/s41593-022-01223-1.

## Supplementary information

Supplementary Information Supplementary Tables 1 and 2.

Reporting Summary

Supplementary Data Editorial assessment report.

## Data Availability

The source data underlying all Figures and Extended Data Figures are provided as Source Data files. All data reported in this study are available from the corresponding authors upon request. Source data are provided with this paper.

## Source data

Source Data Fig. 1 Source data presented in Fig. 1.
Source Data Fig. 2 Source data presented in Fig. 2.
Source Data Fig. 3 Source data presented in Fig. 3.
Source Data Fig. 4 Source data presented in Fig. 4.
Source Data Fig. 5 Source data presented in Fig. 5.
Source Data Fig. 6 Source data presented in Fig. 6.
Source Data Fig. 7 Source data presented in Fig. 7
Source Data Fig. 8 Source data presented in Fig. 8.
Source Data Extended Data Fig. 1 Source data presented in Extended Data Fig. 1.
Source Data Extended Data Fig. 2 Source data presented in Extended Data Fig. 2.
Source Data Extended Data Fig. 3 Source data presented in Extended Data Fig. 3.
Source Data Extended Data Fig. 4 Source data presented in Extended Data Fig. 4.
Source Data Extended Data Fig. 5 Source data presented in Extended Data Fig. 5.
Source Data Extended Data Fig. 6 Source data presented in Extended Data Fig. 6.
Source Data Extended Data Fig. 7 Source data presented in Extended Data Fig. 7.
Source Data Extended Data Fig. 8 Source data presented in Extended Data Fig. 8.
Source Data Extended Data Fig. 9 Source data presented in Extended Data Fig. 9.
Source Data Extended Data Fig. 10 Source data presented in Extended Data Fig. 10.
