# Supplementary material for: Neocortical synaptic engrams for remote contextual memories
Source: Nat Neurosci. 2022 Dec 23;26(2):259–73. doi: 10.1038/s41593-022-01223-1 (PMC9905017; doi:10.1038/s41593-022-01223-1)
Supplement: Supplementary file 1 — Supplementary Tables 1 and 2. [file 41593_2022_1223_MOESM1_ESM.pdf]

nature neuroscience

Article https://doi.org/10.1038/s41593-022-01223-1

Neocortical synaptic engrams for remote contextual memories

In the format provided by the
authors and unedited

SUPPLEMENTARY INFORMATION

Supplementary Table 1 Summary of statistical analysis: t-test*

Data	t-test	Response variable	Groups	DF**	t value	P value
Fig. 1c	Unpaired	Freezing time	HC (10 mice)<br>CFC (11 mice)	17	-1.05	0.308
Fig. 1e	Unpaired	tdTomato+ cell density	HC (8 mice)<br>CFC (7 mice)	6	-3.04	0.023
Fig. 1e	Unpaired	c-Fos+ cell density	HC (8 mice)<br>CFC (7 mice)	12	-1.43	0.179
Fig. 1e	Unpaired	Fos+ proportion among tdT+ cells	HC (8 mice)<br>CFC (7 mice)	12	-6.13	< 0.001
Fig. 1g	Unpaired	tdTomato+ cell density	HC (6 mice)<br>CFC (7 mice)	8	-2.51	0.036
Fig. 1g	Unpaired	c-Fos+ cell density	HC (6 mice)<br>CFC (7 mice)	9	-1.68	0.128
Fig. 1g	Unpaired	Fos+ proportion among tdT+ cells	HC (6 mice)<br>CFC (7 mice)	9	-5.35	< 0.001
Fig. 3f	Unpaired	Freezing time	eYFP (13 mice)<br>MutCREB (15 mice)	25	2.42	0.023
Fig. 3j	Unpaired	AMPAR EPSC	E-E synapses (26 cells)<br>E-NE synapses (24 cells)	47	-1.11	0.273
Fig. 3j	Unpaired	AMPA/NMDA ratio	E-E synapses (32 cells)<br>E-NE synapses (28 cells)	57	-0.88	0.384
Fig. 4i	Paired	qEPSC amplitude	tdT- vs tdT+ mPFC neurons<br>(16 pairs of neurons)	15	0.29	0.776
Fig. 4j	Unpaired	qEPSC (E-E) - qEPSC (E-NE)	7 days after CFC (16 pairs)<br>28 days after CFC (27 pairs)	32	-2.30	0.028
Fig. 5c	Unpaired	tdTomato+ cell density	Casp3- (11 mice)<br>Casp3+ (10 mice)	18	4.18	< 0.001
Fig. 5e	Unpaired	Fos+ proportion among tdT+ cells	Casp3- (8 mice)<br>Casp3+ (9 mice)	13	3.32	0.006
Fig. 5f	Unpaired	Fos+ proportion among tdT+ cells	Casp3- (8 mice)<br>Casp3+ (9 mice)	13	5.09	< 0.001
Fig. 5h	Unpaired	Freezing time	Casp3- (4 mice)<br>Casp3+ (5 mice)	3	7.53	0.005
Fig. 5j	Paired	qEPSC amplitude (DG: Casp3+ group)	tdT- (E-NE) vs tdT+ (E-E) neurons<br>(20 pairs of neurons)	19	-0.04	0.972
Fig. 5j	Paired	qEPSC amplitude (DG: Casp3- group)	tdT- (E-NE) vs tdT+ (E-E) neurons<br>(16 pairs of neurons)	15	-2.92	0.011
Fig. 6k	Unpaired	Freezing time	dCA1: Casp3- (6 mice)<br>dCA1: Casp3+ (6 mice)	6	3.14	0.020
Fig. 6k	Unpaired	Freezing time	RSC: Casp3- (9 mice)<br>RSC: Casp3+ (10 mice)	16	3.41	0.004
Fig. 7e	Unpaired	AMPA/NMDA ratio	E-E synapses (20 cells)<br>E-NE synapses (19 cells)	31	-3.64	0.001

Data	t-test	Response variable	Groups	DF**	t value	P value
Fig. 8g	Paired	AMPA/NMDA ratio	tdT- vs tdT+ mPFC neurons<br>(13 pairs of neurons)	12	-5.00	< 0.001
Ext. Data Fig. 1b	Unpaired	Freezing time	Same context (9 mice)<br>Different context (9 mice)	15	1.95	0.070
Ext. Data Fig. 2e	Paired	AMPA EPSC amplitude	tdT- (NE-NE) vs tdT+ (NE-E) neurons<br>(13 pairs of neurons)	12	-2.24	0.043
Ext. Data Fig. 2e	Paired	AMPA/NMDA ratio	tdT- (NE-NE) vs tdT+ (NE-E) neurons<br>(14 pairs of neurons)	13	-2.68	0.019
Ext. Data Fig. 2h	Paired	AMPA EPSC amplitude	tdT- (NE-NE) vs tdT+ (NE-E) neurons<br>(14 pairs of neurons)	13	-1.09	0.295
Ext. Data Fig. 2h	Paired	AMPA/NMDA ratio	tdT- (NE-NE) vs tdT+ (NE-E) neurons<br>(15 pairs of neurons)	14	-3.82	0.002
Ext. Data Fig. 2k	Paired	AMPA EPSC amplitude	tdT- (NE-NE) vs tdT+ (NE-E) neurons<br>(12 pairs of neurons)	11	-0.15	0.886
Ext. Data Fig. 2k	Paired	AMPA/NMDA ratio	tdT- (NE-NE) vs tdT+ (NE-E) neurons<br>(12 pairs of neurons)	11	0.06	0.954
Ext. Data Fig. 3d	Unpaired	Freezing time	mCherry (9 mice)<br>hM4Di (9 mice)	15	2.68	0.017
Ext. Data Fig. 3e	Unpaired	Freezing time	mCherry (13 mice)<br>hM4Di (10 mice)	20	-0.06	0.952
Ext. Data Fig. 4d	Paired	AMPA EPSC amplitude	tdT- (NE-NE) vs tdT+ (NE-E) neurons<br>(11 pairs of neurons)	10	-1.11	0.293
Ext. Data Fig. 4d	Paired	AMPA/NMDA ratio	tdT- (NE-NE) vs tdT+ (NE-E) neurons<br>(12 pairs of neurons)	11	-1.31	0.219
Ext. Data Fig. 4e	Unpaired	AN (E-E) - AN (E-NE)	Engram inputs (30 pairs)<br>Nonengram inputs (12 pairs)	37	2.92	0.006
Ext. Data Fig. 5e	Paired	AMPAR EPSC	tdT- (E-NE) vs tdT+ (E-E) neurons<br>(16 pairs of neurons)	15	-3.52	0.003
Ext. Data Fig. 5e	Paired	AMPA/NMDA ratio	tdT- (E-NE) vs tdT+ (E-E) neurons<br>(13 pairs of neurons)	12	-4.31	0.001
Ext. Data Fig. 5j	Paired	AMPAR EPSC	tdT- (E-NE) vs tdT+ (E-E) neurons<br>(13 pairs of neurons)	12	-1.52	0.155
Ext. Data Fig. 5j	Paired	AMPA/NMDA ratio	tdT- (E-NE) vs tdT+ (E-E) neurons<br>(13 pairs of neurons)	12	0.00	0.996
Ext. Data Fig. 6d	Paired	qEPSC amplitude	tdT- (E-NE) vs tdT+ (E-E) neurons<br>(20 pairs of neurons)	19	-3.37	0.003
Ext. Data Fig. 6f	Paired	mEPSC amplitude	tdT- vs tdT+ neurons<br>(11 pairs of neurons)	10	-0.42	0.681
Ext. Data Fig. 6i	Paired	qEPSC amplitude	tdT- (E-NE) vs tdT+ (E-E) neurons<br>(14 pairs of neurons)	13	-0.17	0.870
Ext. Data Fig. 7d	Paired	qIPSC amplitude	tdT- vs tdT+ mPFC neurons<br>(24 pairs of neurons)	23	3.35	0.003
Ext. Data Fig. 8c	Paired	AMPA/NMDA ratio	tdT- (NE-NE) vs tdT+ (NE-E) neurons<br>(13 pairs of neurons)	12	-0.20	0.848
Ext. Data Fig. 8g	Paired	qEPSC amplitude	tdT- (E-NE) vs tdT+ (E-E) neurons<br>(20 pairs of neurons)	19	-0.57	0.574

Data	t-test	Response variable	Groups	DF**	t value	P value
**Ext. Data Fig. 8h**	Unpaired	tdTomato+ cell density	Casp3- (5 mice)<br>Casp3+ (6 mice)	4	5.01	0.007
**Ext. Data Fig. 8i**	Unpaired	tdTomato+ cell density	Casp3- (7 mice)<br>Casp3+ (9 mice)	7	7.78	< 0.001
**Ext. Data Fig. 9f**	Unpaired	Freezing time	eYFP (11 mice)<br>PSAM4 (9 mice)	17	2.13	0.048
**Ext. Data Fig. 9g**	Unpaired	Freezing time	eYFP (6 mice)<br>PSAM4 (8 mice)	11	-0.20	0.845
**Ext. Data Fig. 9j**	Paired	AMPA/NMDA ratio	tdT- vs tdT+ BLA neurons<br>(12 pairs of neurons)	11	-3.99	0.002
**Ext. Data Fig. 9k**	Paired	AMPA/NMDA ratio	tdT- vs tdT+ BLA neurons<br>(16 pairs of neurons)	15	-0.73	0.478

* All t-tests were two-sided.
** DF: degree of freedom

Supplementary Table 2 Summary of statistical analysis: ANOVA

Data	ANOVA	Response variable	Factors	DF*	F value	P value
**Fig. 1l**	Repeated measures two-way	Freezing time (%)	Groups	(1,20)	2.74	0.114
			ChR2 (13 mice)			
			eYFP (9 mice)			
			Behavioral session	(1,20)	4.91	0.038
			Laser on			
			Laser off			
			Subject	(20,20)	7.18	< 0.001
			Interaction (group x behavioral session)	(1,20)	9.14	0.007
			** Post hoc comparisons			
			ChR2: laser on versus laser off: P = 0.003			
			eYFP: laser on versus laser off: P = 1.000			
			ChR2 / laser on versus eYFP / laser on: P = 0.021			
**Fig. 2e, 2h**	Two-way	AMPAR EPSC	Test day	(1,92)	17.17	< 0.001
			28 days after CFC (Fig. 2e)			
			7 days after CFC (Fig. 2h)			
			Synapses	(1,92)	8.44	0.005
			E-E synapses			
			E-NE synapses			
			Interaction (test day x synapses)	(1,92)	4.09	0.046
			** Post hoc comparisons			
			28 days after CFC: E-E versus E-NE synapses: P < 0.001			
			7 days after CFC: E-E versus E-NE synapses: P = 1.000			
			E-E synapses: 28 days versus 7 days after CFC: P < 0.001			
**Fig. 2e, 2h**	Two-way	AMPA/NMDA ratio	Test day	(1,93)	0.97	0.327
			28 days after CFC (Fig. 2e)			
			7 days after CFC (Fig. 2h)			
			Synapses	(1,93)	10.08	0.002
			E-E synapses			
			E-NE synapses			
			Interaction (test day x synapses)	(1,93)	8.10	0.005
			** Post hoc comparisons			
			28 days after CFC: E-E versus E-NE synapses: P < 0.001			
			7 days after CFC: E-E versus E-NE synapses: P = 1.000			
			E-E synapses: 28 days versus 7 days after CFC: P = 0.049			
**Fig. 3d**	One-way	AMPA/NMDA ratio	Synapses	(2,46)	10.41	< 0.001
			E-NE synapses (18 cells)			
			E-E/MutCREB- synapses (13 cells)			
			E-E/MutCREB+ synapses (18 cells)			
			** Post hoc comparisons			
			E-NE versus E-E/MutCREB- synapses: P < 0.001			
			E-E/MutCREB- versus E-E/MutCREB+ synapses: P = 0.001			
			E-NE versus E-E/MutCREB+ synapses: P = 1.000			
**Fig. 4e, 4g**	Two-way	EPSC amplitude	EPSC	(1,47)	2.14	0.148
			qEPSC (Fig. 4e)			
			mEPSC (Fig. 4g)			
			Postsynaptic neurons	(1,47)	4.88	0.032
			tdT- neurons			
			tdT+ neurons			
			Cell pairs	(47,47)	3.80	< 0.001
			qEPSC (27 pairs of tdT- / tdT+ neurons)			
			mEPSC (22 pairs of tdT- / tdT+ neurons)			
			Interaction (EPSC x postsynaptic neurons)	(1,47)	4.39	0.042
			** Post hoc comparisons			
			qEPSC: tdT- (E-NE) versus tdT+ (E-E): P = 0.014			
			mEPSC: tdT- versus tdT+: P = 1.000			

* DF: degree of freedom

** Post hoc Bonferroni's simultaneous multiple comparisons

Data	ANOVA	Response variable	Factors	DF*	F value	P value
**Fig. 5d**	Repeated measures two-way	Freezing time (%)	Groups	(1,24)	28.99	< 0.001
			Casp3- (9 mice)			
			Casp3+ (17 mice)			
			Recall session	(1,24)	4.88	0.037
			Recent memory			
			Remote memory			
			Subject	(24,24)	1.55	0.145
			Interaction (group x recall session)	(1,24)	25.73	< 0.001
			** Post hoc comparisons			
			Recent memory recall: Casp3- versus Casp3+: P = 1.000			
			Remote memory recall: Casp3- versus Casp3+: P < 0.001			
**Fig. 7e**	Repeated measures two-way	AMPAR EPSC	BLA neurons	(1,24)	15.30	< 0.001
			tdT- neurons			
			tdT+ neurons			
			Photostimulation intensity	(2,24)	10.56	0.003
			6.3, 13.4, and 20.5 mW/mm^2			
			Cell	(27,24)	7.11	< 0.001
			Interaction (BLA neurons x photostim)	(2,24)	0.41	0.669
**Fig. 7i**	Repeated measures two-way	AMPAR EPSC	mPFC neurons	(1,60)	0.00	0.997
			mCherry- neurons			
			mCherry+ neurons			
			Photostimulation intensity	(3,60)	32.33	< 0.001
			2.8, 6.3, 13.4, and 20.5 mW/mm^2			
			Cell	(20,60)	8.19	< 0.001
			Interaction (mPFC neurons x photostim)	(3,60)	0.09	0.964
**Fig. 8g**	Repeated measures two-way	AMPAR EPSC	mPFC neurons	(1,56)	158.02	< 0.001
			tdT- neurons			
			tdT+ neurons			
			Photostimulation intensity	(2,56)	45.60	< 0.001
			6.3, 13.4, and 20.5 mW/mm^2			
			Cell	(28,56)	30.35	< 0.001
			Interaction (mPFC neurons x photostim)	(2,56)	3.23	0.047
			** Post hoc comparisons			
			6.3 mW/mm^2: tdT- versus tdT+: P < 0.001			
			13.4 mW/mm^2: tdT- versus tdT+: P < 0.001			
			20.5 mW/mm^2: tdT- versus tdT+: P < 0.001			
**Ext. Data Fig. 3c**	Repeated measures two-way	AP firing number	CNO treatment	(1,54)	26.17	< 0.001
			Pre-CNO			
			Post-CNO			
			Current injection	(3,54)	17.84	< 0.001
			100, 200, 300, and 400 pA			
			Cell (9 cells)	(8,54)	4.89	< 0.001
			Interaction (CNO x current injection)	(3,54)	2.2	0.098
**Ext. Data Fig. 10d**	Repeated measures two-way	Freezing time (%)	Groups	(1,13)	24.63	< 0.001
			mCherry (8 mice)			
			hM4Di (7 mice)			
			Recall session	(1,13)	59.50	< 0.001
			Recall 1 (+Tam)			
			Recall 2 (+CNO)			
			Subject	(13,13)	5.71	0.002
			Interaction (group x recall session)	(1,13)	9.51	0.009
			** Post hoc comparisons			
			Recall 1 (+Tam): mCherry versus hM4Di: P = 1.000			
			Recall 2 (+CNO): mCherry versus hM4Di: P < 0.001			
**Ext. Data Fig. 10f**	Repeated measures two-way	Freezing time (%)	Groups	(1,17)	0.00	0.951
			mCherry (9 mice)			
			hM4Di (10 mice)			
			Recall session	(1,17)	4.63	0.046
			Recall 1 (+Tam)			
			Recall 2 (+CNO)			
			Subject	(17,17)	2.19	0.058
			Interaction (group x recall session)	(1,17)	0.01	0.934
