# Supplementary material for: Neocortical synaptic engrams for remote contextual memories
Source: Nat Neurosci. 2022 Dec 23;26(2):259–73. doi: 10.1038/s41593-022-01223-1 (PMC9905017; doi:10.1038/s41593-022-01223-1)
Supplement: Supplementary file 3 — Editorial assessment report. [file 41593_2022_1223_MOESM3_ESM.pdf]

nature portfolio GUIDED OPEN ACCESS

Contents of this report

- **Manuscript details**: overview of your manuscript and the editorial team.
- **Review synthesis**: summary of the reviewer reports provided by the editors.
- **Editorial recommendation**: personalized evaluation and recommendation from all 3 journals.
- **Annotated reviewer comments**: the referee reports with comments from the editors.
- **Open research evaluation**: advice for adhering to best reproducibility practices.

About the editorial process

Because you selected the **Nature Portfolio Guided Open Access option**, your manuscript was assessed for suitability in three of our titles publishing high-quality work across your field of research. More information about Guided Open Access can be found **here**.

Collaborative editorial assessment

Your editorial team discussed the manuscript to determine its suitability for the Nature Portfolio Guided OA pilot. Our assessment of your manuscript takes into account several factors, including whether the work meets the **technical standard** of the Nature Portfolio and whether the findings are of **immediate significance** to the readership of at least one of the participating journals in the Guided OA pilot.

Peer review

Experts were asked to evaluate the following aspects of your manuscript:

- **Novelty** in comparison to prior publications;
- **Likely audience** of researchers in terms of broad fields of study and size;
- **Potential impact** of the study on the immediate or wider research field;
- **Evidence** for the claims and whether additional experiments or analyses could feasibly strengthen the evidence;
- **Methodological detail** and whether the manuscript is reproducible as written;
- Appropriateness of the literature review.

Editorial evaluation of reviews

Your editorial team discussed the potential suitability of your manuscript for each of the participating journals. They then discussed the revisions necessary in order for the work to be published, keeping each journal's specific editorial criteria in mind.

Journals in the Nature portfolio will support authors wishing to transfer their reviews and (where reviewers agree) the reviewers' identities to journals outside of Springer Nature.

If you have any questions about review portability, please contact our editorial office at guidedoa@nature.com.

Manuscript details

Tracking number	Submission date	Decision date
GUIDEDOA-21-00170	24 June 2021	5 October 2021

**Title**	Neocortical Synaptic Engrams for Remote Contextual Memories	**Corresponding author**	Jun-Hyeong Cho<br>**Affiliation:** University of California, Riverside
**Preprint information**	There is no preprint posted for this manuscript.	**Peer review type**	Single-blind

Editorial assessment team

**Primary editor**	**Sachin Ranade**<br>**Home Journal:** *Nature Neuroscience*, ORCID: 0000-0002-5150-5776<br>**Email:** sachin.ranade@us.nature.com
**Editorial team members**	**David Rowland**, *Nature*, ORCID: 0000-0002-2735-2730<br>**Christian Schnell**, *Nature Communications*, ORCID: 0000-0002-3499-9217
**About your primary editor**	Sachin received his PhD from Stony Brook University for his work on the responses of serotonin neurons in the dorsal raphe nucleus of rats engaged in an olfactory perceptual decision task. During his postdoctoral research at Cold Spring Harbor Laboratory he developed optogenetic tagging techniques to identify neural activity from distinct neuronal subtypes in mice to investigate their behavioral correlates. Prior to joining Nature Neuroscience, he was an editor at Nature Communications. His research interests include neural mechanisms of behavioral function and dysfunction at the circuit, systems, and computational level. Sachin is based in the New York office.

Editorial assessment and review synthesis

**Editor's summary and assessment**	The manuscript presents a detailed circuit characterization of the synaptic strengthening of engrams in mPFC and other areas during the memory consolidation phase. The study uses multiple approaches including activity dependent inducible Fos labeling, circuit tracing, optogenetics, and slice physiology. The engram circuit studied here includes the following projections: DG -> Retrosplenial ctx -> mPFC, recurrent mPFC engram circuit, and mPFC -> BLA.<br><br>Contextual memories involve hippocampal circuits during acquisition, and these are stabilized through consolidation in the cortex. Memory acquisition involves synaptic plasticity [1, 2] and we know that mPFC and ACC engrams are involved in consolidation [9-12]. Ref 9 showed the dynamics of engrams in the hippocampus, cortex and BLA during consolidation. This paper extends this work by identifying a multi-area circuit with connected engrams that undergo synaptic strengthening. Some experiments appear underpowered and some effects seem to be weak.
**Editorial synthesis of reviews**	Your manuscript has been seen by 3 reviewers with overlapping expertise in synaptic and circuit mechanisms underlying memory processes including memory engrams.<br><br>All reviewers appreciated the comprehensive nature of the analyses and there were no issues raised regarding the novelty of these findings. The reviewers identify a number of concerns that need to be addressed with additional experiments in order to strengthen the existing findings through the addition of various control conditions to address potential confounds, replicates to improve reproducibility, and silencing experiments to test for causal involvement. These experiments are necessary to provide a more complete picture regarding the behavioural significance of the various engrams interrogated in this work.<br><br>As part of the Guided Open Access pilot, editors from *Nature, Nature Neuroscience* and *Nature Communications* have discussed the reviewer reports and the manuscript's suitability for our journals. After careful evaluation, our editorial recommendation is to revise the manuscript and submit back through the Guided Open Access submission portal for consideration at *Nature Neuroscience* or *Nature Communications*.

Editorial recommendation

Journal	Recommendation	Comment
Nature	Revision not invited	Following editorial assessment of the paper and reviewer reports it was felt that the conceptual advance is not sufficient for further consideration at Nature
Nature Neuroscience	Major revisions with extension of the study	The reviewers have raised important concerns regarding the functional significance of the various circuit elements studied in the manuscript w.r.t their role in systems consolidation. The work also requires additional experiments for reproducibility, robustness and completeness. The editors feel that these concerns can potentially be addressed in a major revision with substantial additional experimentation.
Nature Communications	Major revisions	The reviewers have raised important concerns regarding the functional significance of the described various circuit elements in systems consolidation. The editors at Nature Communications feel that addressing the concerns about reproducibility, robustness and completeness can be addressed in a major revision for publication in Nature Communications.

Next steps

Recommendation Summary

- Revise for consideration at Nature Neuroscience
- Revise for consideration at Nature Communications

See the previous page for details.

Revision

To follow our recommendation, please upload the revised manuscript, along with your point-by-point response to the reviewers' reports and editorial advice **using the link provided in the decision letter**.

Revision checklist

- Cover letter, stating to which journal you are submitting
- Revised manuscript
- Point-by-point response to reviews
- Updated **Reporting Summary** and **Editorial Policy Checklist**
- Supplementary materials (if applicable)

Submission elsewhere

Within the Nature Portfolio

Springer Nature provides authors with the ability to transfer a manuscript within the Nature Portfolio, without the author having to upload the manuscript data again. To use this service, please **follow the transfer link provided in the decision letter**.

Note that any decision to opt in to *In Review* at the original journal is not sent to the receiving journal on transfer. You can opt in to *In Review* at receiving journals that support this service by choosing to modify your manuscript on transfer.

To a journal outside of Nature Portfolio

If you choose to submit your revised manuscript to a journal at another publisher, we can share the reviews with another journal outside of the Nature Portfolio if requested. You will need to request that the receiving journal office contacts us at guidedOA@nature.com. We have included editorial guidance below in the reviewer reports and open research evaluation to aid in revising the manuscript for publication elsewhere.

Annotated reviewer reports

The editors have included some additional comments on specific points raised by the reviewers below, to clarify requirements for publication in the recommended journal(s). However, please note that all points should be addressed in a revision, even if an editor has not specifically commented on them.

Reviewer #1

**Reviewer #1**	This reviewer has not chosen to waive anonymity. The reviewer's identity can only be shared with representatives of an established journal editorial office.
**Reviewer #1 expertise**<br>Summarised by the editor	Circuit mechanisms of memory, engrams
**Editor's comments about this review**	This reviewer is overall skeptical of the advance presented in these findings. They have identified a number of important experiments that need to be addressed with additional experiments to improve the overall impact of the findings. They also have concerns about the experimental design regarding the timing of the synaptic strengthening with respect to the remote recall test.

Reviewer #1 comments

**Overview**	Remarks to the Author: Overall significance:<br>Lee et al. investigate synaptic strengthening of prefrontal (prelimbic cortex) contextual fear conditioning (cFC) engrams at 7d and 28d after learning. In most experiments, this involves remote fear recall at 28d (or 7d), followed by electrophysiology of connected engram and non-engram neurons. The engram neurons are defined upon tagging experiments in cFos-CreERT2 knock-in mice.<br>The main findings are that inter-hemispheric and (to a much lesser extent) local recurrent excitatory connections between engram neurons are strengthened at 28d but not 7d. Expression of a dominant-negative CREB construct in the contralateral hemisphere prevents strengthening (but here not tested upon recall). Likewise, extinction of remote memory using a 5-day protocol leads to reduced fear recall and no strengthened connections between mPFC engram neurons. Ablation of dorsal dentate gyrus engram neurons a few days after learning suppresses remote recall as well as strengthening of mPFC engram neuron connections. In addition, engram neurons in RSC receive connection from engram neurons in dCA1 and connect to engram neurons in mPFC, and engram neurons in mPFC connect directly and

indirectly to engram neurons in BLA. Finally, mPFC engram neurons labeled at learning connect to additional cFos+ neurons in mPFC upon remote recall.

This study provides a comprehensive analysis of remote cFC engram neurons in mPFC, and the synaptic strength of their connections at early (7d) and late (28d) recall. The analysis is of good technical quality, and the findings are consistent with a number of previous claims about remote fear memory, adding a synaptic and engram dimension to those claims. However, the study has major weaknesses that greatly detract from its potential value (see points below). Briefly, key weaknesses include absence of causal evidence for most findings, and a major confound related to strengthening induced upon remote recall as opposed to strengthening already present before remote recall (i.e. "systems consolidation" of the mPFC engram). Due to these limitations, the advance provided by this study is modest.

Specific comments

#	Reviewer comment	Editorial comment
1	Most evidence in the study is based on gain-of-function experiments; what is missing is evidence that silencing the mPFC engram neurons interferes with remote fear memory recall (but not early recall at 7d). This might have been shown for some previous studies, but needs to be shown under the conditions of this study.	
2	The authors need to elucidate the issue of whether strengthening reflects plasticity induced upon remote recall, as opposed to "systems consolidation" plasticity induced during the weeks preceding remote recall. This will require experiments carried out without behavioral remote recall and comparison of the outcomes under those different conditions. If the plasticity is induced by recall, the significance of these findings changes substantially, and the results can't be used to argue about direct mechanisms of systems consolidation.	
3	The dnCREB results were obtained without remote recall. They need to be complemented by remote recall experiments in order to compare to the other data in the study.	Nature Neuroscience and Nature Communications will both require you to address the comments #1-#3 with additional experiments to strengthen the study.
4	The significance of the findings in Figs. 6 and 7 (RSC, BLA) is unclear since the data only address anatomical	Nature Neuroscience will require you to extend the findings by

	connectivity. Claims of possible functional significance are not warranted.	performing functional significance experiments for RSC, BLA
**5**	The DG ablation experiments are convincing but they can't be used in their current form to argue that activity in hippocampal engrams is required for prefrontal engram strengthening – the experiment was done upon remote recall, which is inhibited by the ablation procedure.	
**6**	Synaptic strengthening in recurrent collaterals of mPFC engram neurons is weak at best. Given that the behavioral significance of inter-hemispheric strengthening is unclear, it is not clear to what extent the synaptic strengthening data can be extrapolated to function of the mPFC engram in remote recall.	
**7**	Minor:<br><br>1) Freezing induced in context B upon mPFC engram neuron activation is very variable and generally weak. It would be useful to also present average values, with their error bars.	

**Reviewer #2**	
**Reviewer #2**	This reviewer has not chosen to waive anonymity. The reviewer's identity can only be shared with representatives of an established journal editorial office.
**Reviewer #2 expertise**<br>Summarised by the editor	Circuit mechanisms of memory, engrams
**Editor's comments about this review**	The reviewer has provided an overall positive assessment of the paper, but please see major comments #1-#3 and #5.
**Reviewer #2 comments**	

**Overview**	Memories for episodic-like information/events are thought to be encoded in hippocampal-cortical networks. At recent time points, the hippocampus is essential for retrieval of these memories. However, at more remote delays, cortical regions, including the mPFC, play increasingly important roles in their retrieval. The current paper by Lee and colleagues explores the evolving role of mPFC engram cells 9and their connections) in these memories using viral tracing, engram tagging, behavioral and patch-clamp recording methods. They present several new and exciting findings, the most novel of which I highlight here: 1) excitatory connectivity between mPFC engram neurons is increased at the remote delay. This is observed both within and between hemispheres, and is the first observation of stably potentiated synapses (measured by EPSCAMPAR and AMPA/NMDA ratio) at this remote delay; 2) extinction at remote time points reverses these learning-induced changes; 3) intra-hemispheric strengthening of mPFC engram circuitry depends on activity of DG engram cells; 4) expansion of mPFC engram circuitry at remote time-points involves recruitment of mPFC 'recall' neurons that are monosynaptically connected to mPFC engram neurons (tagged at the time of training).<br><br>This is a very nice study. It presents lots of intriguing, novel findings and advances our understanding of systems consolidation.<br><br>Remarks to the Author: Impact:<br><br>This is a really fantastic paper. With appropriate revisions, i think this would be a good candidate for Nature Neuroscience.

Specific comments

#	Reviewer comment	Editorial comment
1	Remarks to the Author: Strength of the claims:<br><br>Major:<br><br>Recall vs. Relay neurons. The authors suggest that synaptic strength between mPFC engram cells (i.e., E-E) is significantly enhanced compared to that between mPFC engrams and non-engram cells (i.e., E-NE and NE-E; Figure 2, Figure 4, and Extended Data Figure 3). However, the authors also suggest that mPFC engram cells exhibit enhanced synaptic strength with 'mPFC relay neurons' (Figure 7) and 'mPFC recall neurons' (Figure 8), which are presumably non-engram populations. Can the authors clarify this discrepancy. Also, to what extent do these two	Nature Neuroscience and Nature Communications will both require you to address this concern

	populations (recall vs. relay) overlap? Are recall neurons relay neurons? And vice-versa?	
**2**	Recall vs. relay neurons. These subpopulations are among the most interesting findings in the paper. However, we don’t have any functional insights into their roles in memory retrieval, particularly at remote time points. For example, does inhibiting relay neurons (or mPFC relay projections) impact memory expression? Is it possible to selectively inhibit recall neurons? What if recall neurons labeled at recent time points (i.e., non-engram neurons) are inactivated?	Nature Neuroscience will require you to address this concern with new experiments.
**3**	Controls. In several experiments controls are missing. For example, for the mCREB (Fig. 3) expts, there are no mCREB- controls. For the extinction experiments (Fig. 4), there are no ‘no extinction’ groups. For the DG engram cell killing experiment (Fig. 5) there are no ‘no killing’ controls. And so on. I appreciate to some extent why the authors chose this strategy—they are contrasting the findings presented in these figures with the potentiation observed in Figs. 2 and 4. However, there are a couple of issues with this. On one hand, these subsequent experiments might represent simple failures to replicate the initial effects (i.e., they have nothing to do with the various viral or behavioral manipulations). On the other hand, the absence of enhanced synaptic strength might be a consequence of some non-specific aspect of their viral intervention that can only be controlled for by having non-mCREb expressing controls. I don’t doubt the findings, but having the basic effects replicated across (at least some) experiments I think is critical to increase confidence.	Nature Neuroscience and Nature Communications will both require you to address this concern
**4**	Introduction and discussion. The results are well-presented. However, I think the introduction and discussion need some work. The introduction is a little repetitive—appealing too frequently to the idea that synapse-strength has not been assessed in the context of systems consolidation. Perhaps the authors can foreshadow some of the other interesting findings (relay cells, recall cells etc) and relate these questions to theoretical models of systems consolidation (e.g., transformation theory, standard model). The discussion reads a little too much as a repetition of the results—again	

	discussing these findings in the context of current views of systems consolidation will better contextualize this interesting work.	
5	DG vs. CA1. The authors investigated whether and how the mPFC engrams receive mnemonic signals from hippocampal engrams. To this end, the authors ablate DG engrams and measure the changes of synaptic strength in mPFC engrams (Figure 5). Next, the authors investigated how the dorsal hippocampal CA1 engrams send outputs to the mPFC engrams (Figure 6). Here, there is a leap of logic. Although CA1 is thought of as a major output of hippocampal subregions, DG-CA3 also projects to cortical regions. This makes the authors finding unclear. Specifically, it is unclear whether the CA1-mPFC circuit is important for the functional development of the mPFC engrams due to the absence of data on CA1 engram-mediated intervention of the mPFC engram synaptic strength (Figure 6).	Nature Neuroscience and Nature Communications will both require you to address this concern, which is an overlapping concern from all reviewers.
6	Minor:<br>1. Lack of memory generalization. With time, context memories typically generalize (i.e., exhibit equivalent freezing in training vs. novel context). The authors fail to see this time-dependent emergence of generalization (which is an important hallmark of systems consolidation, and relevant to transformation theory etc). Perhaps the authors can speculate why they don't see this—are the contexts very distinct (more so than would be typically used)? Were context presentations counter-balanced? If not, might this reflect extinction?<br>2. Rationalization for some experiments. The authors should provide stronger rationales for experimental choices in various places. For example, why focus inter-hemispheric engram-engram synapse strength vs. other inputs into mPFC? In the DG engram cell killing experiment, why focus on intra-hemispheric (vs. inter-hemispheric) connectivity? For the RSC experiments, why shift to CA1? Obviously, this is because of the anatomy, but the earlier killing experiment ablated DG engram cells, rather than CA1 engram cells.<br>3. In the ChR2-mediated reactivation of mPFC engram test (Figure 1i to l), the authors test the same mice	

	for 9 minutes each for 3 consecutive days (total 27 minutes). The amount of total retrieval time is comparable to the authors' extinction protocol (total 30 minutes through 5 days; Figure 3). Can the authors comment on why there were 3 optogenetic reactivation test tests?<br><br>4. In Figure 5, the authors test the same mice at both recent and remote time points. This introduces confounds (reconsolidation/extinction etc) that complicate interpretation of the data.<br><br>5. Extinction. The result that extinction modifies engram cells—labeled at the time of training—is quite striking and important yet is barely discussed. The general consensus is that extinction involves the formation of an inhibitory 'no CS' memory that competes with the original engram. In general, researchers favor the idea that the original engram remains largely intact (evidence for this being renewal, reinstatement etc). The finding that the original engram appears to be modified by extinction training is therefore quite surprising in this regard, and deserves highlighting.	
**7**	Remarks to the Author: Reproducibility:<br><br>The core findings-- potentiated inter-hemispheric and intra-hemispheric connectivity between mPFc engrams neurons are only presented once. Ideally subsequent experiments should build in replication of these effects-- they do not because they lack appropriate controls (see review above).	

**Reviewer #3**	
**Reviewer #3**	This reviewer has not chosen to waive anonymity. The reviewer's identity can only be shared with representatives of an established journal editorial office.
**Reviewer #3 expertise**<br><br>Summarised by the editor	Circuit mechanisms of memory, engrams
**Editor's**	This reviewer provides prescriptive suggestions to improve the robustness of the

**comments about this review**	findings. They also suggest that the manuscript requires some re-writing to provide a cohesive framework.

Reviewer #3 comments

**Overview**	Remarks to the Author: Overall significance: The study of Lee et al. provides a large number of interesting results on synaptic mechanisms of system-level memory consolidation. Taken separately, the different experiments contain interesting pieces of evidence on these mechanisms. However, the whole study is missing a cohesive framework, where tracking similar variable within the different synapses analyzed, would allow to compare the results across experiments. This lack of coherent framework prevents the authors to draw a complete picture with their findings.

Specific comments

#	Reviewer comment	Editorial comment
**1**	Remarks to the Author: Impact: Specifically, one of the most important claim of the study is the time specificity of the engram to engram (E-E) mPFC-contra.mPFC synapses, which is potentiated after 28 days, but not after 7 days. A refined timeline of the development of this plasticity would strongly increase the impact of their conclusions.	Nature Neuroscience will require you to explore the timeline of synaptic strengthening further.
**2**	From Figure 1-3 the main quantified variable is input specific AMPA/NMDA. However, from Figure 4-8, the authors then quantify qEPSC and mEPSC. While interesting, it is important to provide the changes in AMPA/NMDA in mPFC-contra.mPFC synapses after the different manipulations, including the DG engram cell ablation: for example in Figure 5 the authors deleted the DG engram and then measures qEPSCs. Recording the AMPA/NMDA ratio of the mPFC-contra.mPFC synapses would have been more informative to support the initial observation that mPFC-contra.mPFC synapses potentiation encode long term CFC memory.	Nature Neuroscience and Nature Communications will both require you to address this concern.
**3**	While manipulating the DG engram is interesting, the dCA1-mPFC synapses have been well described, and assessing the evolution of the strength of these synapses	

	after CFC would also strengthen the study and support the system model of the authors. Thus, in Figure 6, it remains unclear why the authors focused on the RSC rather than the dCA1, or the contralateral mPFC. Especially as the RSC-contraRSC synapses do not display plasticity after CFC (AMPA/NMDA, extended Figure 5).	
**4**	Remarks to the Author: Strength of the claims:<br><br>The most important and interesting finding of the study is the time and pathway specificity of synaptic plasticity, strongly supporting a cortical system-level mechanism for memory consolidation.<br><br>However, the authors should be careful to not over-interpret their results in the discussion. For example, Line 341: “the permanent storage of contextual memories”: the authors did not test permanent storage, and only tested it up to 28 days, which represents less than 4% of the life of a mouse. Thus, the authors should temper this sentence.	
**5**	Minor comments<br><br>Please add the first study recording engram-engram synaptic strength with AMPA/NMDA ratio: Ryan et al. 2017 PMID: 26023136<br><br>Extended Figure 1d: add ‘among all *layers* tdT+ mPFC neurons’ in the y axis title<br><br>Figure 2a & Extended Figure 3a: on the right diagram, please add PFC and contra-PFC on the top of the neurons.	
**6**	Remarks to the Author: Reproducibility:<br><br>Regarding the ex vivo recordings of AMPA/NMDA, the exact delay between the recall test and decapitation of the mice for slice electrophysiology needs to be specified. Although I could not find this information in the manuscript, I assume the delay was minimal, in order for the recall session to not induce plastic changes. An	Nature Neuroscience will require you to address this concern, which overlaps with Reviewer 2.

	additional experiment quantifying the AMPA/NMDA 24h after the test session could support the implication of this set of synapses in memory retrieval.<br><br>The amplitude of the engram cells inputs (E-E and E-NE) EPSCs is very small (less than 30 pA) and therefore noisy. The authors should provide data to illustrate the stability (variability of amplitude in % of average over repeated stimulations) of the EPSCs amplitude over repeated stimulations, as well as the failure rate.	

Open research evaluation

Data availability
**Data availability statement**
Thank you for including a Data Availability statement. However, we noted that you have only indicated that data are available upon request. The data availability statement must make the conditions of access to the "minimum dataset" that are necessary to interpret, verify and extend the research in the article, transparent to readers.<br><br>In addition, Nature Portfolio policies include a strong preference for research data to be archived in public repositories. For data types without specific repositories, we recommend that data are deposited in a generalist repository such as figshare or Dryad. More information about our data availability policy can be found here: https://www.nature.com/nature-portfolio/editorial-policies/reporting-standards#availability-of-data<br><br>See here for more information about formatting your Data Availability Statement: http://www.springernature.com/gp/authors/research-data-policy/data-availability-statements/12330880
**Other data requests**
All source data underlying the graphs and charts presented in the main figures must be made available as Supplementary Data (in Excel or text format) or via a generalist repository (eg, Figshare or Dryad). This is mandatory for publication in a Nature Portfolio journal, but is also best practice for publication in any venue.
**Reporting & reproducibility**
We encourage you to share your step-by-step experimental protocols on a protocol sharing platform of their choice. The Nature Portfolio's Protocol Exchange is a free-to-use and open resource for protocols; protocols deposited in Protocol Exchange are citable and can be linked from the published article. More details can be found at www.nature.com/protocolexchange/about
When choosing a color scheme please consider how it will display in black and white (if printed), and to users with color blindness. Please consider distinguishing data series using line patterns rather than colors, or using optimized color palettes such as those found at https://www.nature.com/articles/nmeth.1618 The use of colored axes and labels should be avoided. Please avoid the use of red/green color contrasts, as these may be difficult to interpret for colorblind readers.

We have included as an attachment to the decision letter a version of your Reporting Summary with a few notes. This is mainly for your information, but we hope it is helpful when preparing your revised manuscript. If you decide to resubmit the manuscript for further consideration, please be sure to include an updated Reporting Summary.
